# Direct and Semi-Direct Composite Techniques in Posterior Teeth: A Two-Year Follow-Up Comparative Study

**DOI:** 10.3390/jcm15020687

**Published:** 2026-01-14

**Authors:** Adriana Saceleanu, Anca Maria Fratila, Vasile Calin Arcas, Cristina Ana-Maria Arcas, Dragos Anton Dadarlat, Laura Stef

**Affiliations:** 1 Department of Dental Medicine and Nursing, Faculty of Medicine, Lucian Blaga University of Sibiu, 550169 Sibiu, Romania; adriana.saceleanu@ulbsibiu.ro (A.S.); dragos.dadarlat@ulbsibiu.ro (D.A.D.); laura.stef@ulbsibiu.ro (L.S.); 2 Military Clinical Emergency Hospital, 550024 Sibiu, Romania; 3 Dental Medicine Research Center, Faculty of Medicine, Lucian Blaga University of Sibiu, 550169 Sibiu, Romania; 4 Doctoral School, Faculty of Medicine, Lucian Blaga University of Sibiu, 550169 Sibiu, Romania; 5 Department of Radiology and Medical Imaging, Clinical Emergency County Hospital of Brasov, 500326 Brasov, Romania; arcascristina@yahoo.com

**Keywords:** dental restoration, permanent, tooth, posterior, dental aesthetics, clinical evaluation, dental marginal adaptation, dental polishing, dental wear

## Abstract

***Background:*** Composite restorations are the standard of care for posterior teeth due to their aesthetic properties and conservative nature. However, the choice between direct and semi-direct techniques can influence clinical longevity and performance. ***Objectives:*** This study aimed to compare the clinical performance of two restorative approaches: a direct technique and the semi-direct onlay technique in terms of aesthetic quality, surface finish, wear resistance, marginal integrity, and overall clinical efficiency over a two-year period. ***Methods:*** A total of 348 composite restorations were placed in 192 patients. Each restoration was evaluated at four timepoints: baseline (T0), 6 months (T1), 1 year (T2), and 2 years (T3). Clinical performance was assessed using standardised 5-point rating scales across the five dimensions. Repeated-measures ANOVA assessed changes over time, while Wilcoxon signed-rank and Mann–Whitney U tests were used for intra- and inter-group comparisons. ***Results***: Significant time effects were observed across all clinical parameters (*p* < 0.0001). The direct technique exhibited superior initial results in aesthetics and surface finish at T0 and T1 (*p* < 0.001), but differences diminished by T3. In contrast, the semi-direct technique demonstrated improved performance in wear resistance and marginal integrity at T2 and T3. Both techniques showed progressive deterioration, particularly in marginal adaptation. ***Conclusions:*** The direct technique offers enhanced short-term aesthetics and procedural efficiency, while the semi-direct approach provides superior long-term durability and marginal adaptation.

## 1. Introduction

Composite restorations in posterior teeth represent a cornerstone in restorative dentistry [1], aimed at maintaining both functional integrity and aesthetic outcomes [2]. The placement of these restorations must withstand significant masticatory forces while simultaneously providing a lifelike appearance that blends with natural dentition [3]. Traditionally, restorative techniques for posterior teeth have evolved to enhance the durability, appearance, and efficiency of these procedures [4,5]. Among the numerous approaches, the direct composite technique (such as Stamp Technique, Layering Technique, Anatomical Direct Restoration) and the semidirect onlay technique stand out for their respective advantages in different clinical contexts.

Among the direct composite techniques, Stamp Technique is a restorative approach [6] that allows clinicians to replicate the original occlusal anatomy of posterior teeth with impressive accuracy [6,7,8]. This method involves shaping the composite material directly in the cavity to mimic the tooth’s natural contours [7,9]. After the cavity is prepared and decay is removed [10,11], the composite is placed and sculpted, ensuring a close replication of the tooth’s original anatomy [7,9,12]. The direct composite techniques are particularly effective for small- to medium-sized Class I and Class II cavities [6,8,12,13], where much of the natural tooth anatomy is preserved [14]. Its primary advantage lies in its ability to reduce the amount of time needed for occlusal adjustments post-placement [15] while improving the accuracy of the restoration [8,12].

On the other hand, the semidirect onlay technique is a method that involves creating a restoration outside of the mouth using composite resin [16,17], which is then bonded to the prepared tooth [18]. This technique is useful for larger restorations or when a more robust structure is required, particularly in cases where significant tooth structure has been lost [19]. The semidirect approach allows for better control over the occlusal morphology and contact points [20,21], as the restoration is fabricated with precision in the dental office before final cementation [22]. The ability to work outside the oral cavity offers an opportunity for improved curing [23], minimising polymerization shrinkage [24] and ensuring a more durable restoration [25].

The direct composite technique offers the advantage of replicating the pre-existing occlusal anatomy, either by using an occlusal matrix, such as in the stamp technique, or by guiding the restoration to the remaining cusp tissue anatomy, as seen in the anatomical direct restoration and layering technique [7], often minimising adjustments post-placement [26]. However, the necessity to ensure perfect marginal closure and avoid open margins remains [27], a challenge with direct techniques, particularly due to polymerization shrinkage [12,28]. This can require additional chairside adjustments to achieve a precise fit [26]. The semidirect onlay technique, on the other hand, offers better control over these functional aspects [29], allowing clinicians to create restorations with more accurate marginal seals [30] and occlusal morphology [31], which reduces the need for extensive post-cementation adaptation [29,30].

When considering composite restorations for posterior teeth, several critical aspects influence the success and longevity of the procedure. Aesthetic outcomes, such as colour matching and surface smoothness, are essential for patient satisfaction, as composites must blend seamlessly with the natural tooth [32]. Functional outcomes, including proper occlusion [33] and marginal integrity [27], ensure that the restoration can withstand masticatory forces without causing discomfort or further damage to adjacent structures. Additionally, wear resistance plays a pivotal role in maintaining the durability of the restoration over time [34], while marginal closure and microleakage control are vital to preventing secondary caries [35]. Surface finish also impacts plaque accumulation and the long-term aesthetic stability of the restoration [36]. Lastly, the efficiency and time taken for the procedure, particularly in chairside techniques, affect the overall treatment experience for both the patient and clinician [37].

Achieving both functional and aesthetic success in posterior composite restorations is often challenging due to the complex anatomy of these teeth, which requires careful attention to occlusal relationships, contact points, and overall tooth morphology [15,38]. Beyond restoring function, aesthetics play an increasingly significant role in patient satisfaction, particularly with composite materials that closely mimic the appearance of natural enamel and dentin [32]. For practitioners, the balance between aesthetics and function is paramount, as these two factors are interrelated and must be considered in any restorative approach [39,40].

Proper occlusal relationships are crucial to ensure that the restoration harmonises with the patient’s bite and avoids interference with neighbouring and opposing teeth [41]. In both the direct composite and semidirect onlay techniques, accurate occlusion is key to preventing issues like premature wear or discomfort [42,43].

Wear resistance is a critical factor for posterior restorations, as these teeth are subjected to higher masticatory forces compared to anterior teeth [34]. The durability of composite materials has been a point of concern [44], especially with direct techniques, where polymerization shrinkage and the inability to fully cure the material can compromise longevity [12,28,45]. Studies suggest that semidirect onlays exhibit superior wear resistance over time [46] compared to direct composite techniques [12], largely due to the controlled conditions under which the restoration is fabricated [47].

Surface finish is another important consideration [36], as a smoother surface reduces plaque accumulation [48] and enhances the long-term aesthetic appearance of the restoration [44,49]. While both techniques can achieve excellent surface finishes [6,9,50], the semidirect method allows for more controlled polishing and finishing outside the mouth, leading to superior smoothness [50] compared to direct composite restorations [9,51]. In contrast, the direct technique, while efficient, may require additional finishing due to the challenges of achieving a clinically acceptable occlusal smoothness [9].

Marginal integrity and microleakage are also pivotal concerns for the longevity of composite restorations. Poor marginal integrity can lead to microleakage, which in turn increases the risk of secondary caries [52]. The semidirect technique often provides better marginal adaptation and reduces the risk of microleakage due to the controlled setting in which the restoration is crafted and polymerized [27,35]. However, with careful execution, the direct technique can still offer satisfactory marginal integrity, especially in cases where minimal tooth structure is involved [27,30].

Time and efficiency are critical considerations for both clinicians and patients [4,5,37]. The direct composite technique is renowned for its rapid execution and simplicity, often requiring less chairside time than more labour-intensive methods like the semidirect technique [7,53,54]. However, the latter offers the advantage of reduced need for refinements post-placement, potentially saving time in complex cases despite the additional steps required during the fabrication process [55].

While both the direct composite and semidirect onlay techniques are well established in restorative dentistry, there is a lack of comparative clinical data evaluating their long-term performance across standardised functional and aesthetic criteria in real-world settings.

Given these considerations, the aim of this study is to directly compare the direct composite techniques and the semidirect onlay technique for posterior composite restorations in terms of aesthetic outcomes, wear resistance, surface finish, marginal integrity, microleakage, time, and efficiency. Additionally, the study seeks to explore whether a hybrid approach, combining the best aspects of both techniques, can be developed to optimise both functional and aesthetic outcomes in posterior restorations. Based on their individual characteristics, the authors hypothesise that while the direct composite technique will demonstrate superior aesthetic outcomes and procedural efficiency in the short term, the semidirect onlay technique will offer greater long-term durability, wear resistance, and marginal integrity.

The novelty of this study lies in its real-world, two-year clinical comparison of two widely used restorative techniques, direct composite and semidirect onlay, using standardised evaluation criteria to assess their functional and aesthetic performance over time. Unlike prior case reports or in vitro studies, this investigation provides longitudinal clinical evidence from a substantial patient cohort. Based on this approach, the null hypothesis tested was that no statistically significant differences would be observed between direct and semi-direct composite restorations in terms of clinical performance during the follow-up period.

## 2. Materials and Methods

### 2.1. Study Design

This clinical trial was conducted by researchers from the Lucian Blaga University of Sibiu in collaboration with the Military Hospital of Sibiu. The trial aimed to compare the outcomes of composite restorations using two distinct techniques, a semidirect technique suited to the case and a semidirect onlay technique. The study spanned two years from April 2022 to September 2024, and the restorations were realised from April 2022 to September 2022, during which a total of 348 composite restorations were performed on 192 patients. The patients were selected based on the need for posterior composite restorations, with specific inclusion and exclusion criteria to ensure consistency in treatment application.

Patients received follow-up evaluations at intervals of 6 months, 1 year, and 2 years post-treatment to assess the long-term outcomes of their restorations. These follow-ups were designed to monitor various parameters, including aesthetic outcomes, wear resistance, surface finish, marginal integrity, and microleakage. Each follow-up evaluation involved clinical examinations, radiographic assessments, and patient feedback to measure functional and aesthetic outcomes over time. All the procedures were performed by a single calibrated operator to eliminate inter-operator variability. The same person performed the evaluation. The study was designed to provide a robust comparison between the two techniques.

### 2.2. Inclusion and Exclusion Criteria

The inclusion criteria for this study ensured that the participant population would provide consistent, relevant data on posterior composite restorations. Only patients between 18 and 65 years of age were included, as this demographic generally has fully developed dentition and consistent masticatory forces. All patients needed Class I or Class II carious lesions in their posterior teeth that required composite restorations, ensuring that the study focused on relevant dental treatments. Additionally, patients were required to demonstrate good oral hygiene with minimal periodontal disease, as poor oral hygiene could have skewed the results by introducing confounding factors related to plaque accumulation. Another criterion was stable and functional occlusion, which would ensure that post-treatment occlusal adjustments wouldn’t affect the study outcomes. Participants were also excluded if they had allergies to the composite resin, adhesive, or any other dental materials used in the study. Finally, all participants were required to provide informed consent and commit to attending all follow-up visits over the two-year duration of the study.

The exclusion criteria were designed to eliminate patients who could potentially introduce variables that might compromise the study results. Individuals with systemic health conditions that could affect oral health or the healing process, such as uncontrolled diabetes or autoimmune diseases, were excluded to maintain the focus on the dental treatments themselves rather than underlying health issues. Pregnant women were also excluded to avoid any potential risks associated with dental materials or procedures during pregnancy. Patients with bruxism or other parafunctional habits were excluded due to the increased likelihood of restoration failure caused by excessive occlusal forces. Additionally, patients with significant tooth loss or compromised tooth structure, such as those requiring full crowns, were not included, as the study was focused on more conservative composite restorations.

To ensure uniformity in the condition of the teeth being treated, patients with pre-piously root canal-treated teeth were excluded from the study, as these teeth often exhibit compromised structural integrity. Finally, patients who failed to attend the follow-up visits or were unable to commit to the 2-year follow-up schedule were also excluded from the final analysis. This ensured that only reliable data from consistent follow-ups would be used to evaluate the outcomes of the two techniques. These criteria allowed the researchers to control for variables that could otherwise impact the comparison between the direct and semidirect onlay techniques.

### 2.3. Sample Size and Groups

The study involved a total sample size of 192 patients, who collectively received 348 composite restorations over the six-month treatment period. These patients presented with carious lesions in their posterior teeth, and the treatments were distributed between two techniques: 147 restorations were performed using the direct techniques, of which the most common was the stamp, while 201 restorations were carried out using the semidirect onlay technique. The final data analysis included 343 restorations as out of the initial 192 patients, three did not return for follow-up appointments, resulting in the exclusion of five restorations from the final analysis, two from the direct restoration group and three from the onlay technique group. The rationale for selecting a specific direct composite technique was based on the well-established indications for each technique, as recognised by clinicians, and their professional judgement. This study does not aim to determine which direct technique is superior to others, as such comparisons have been extensively studied and the results are well-known. Instead, the focus of this study is on comparing direct and semi-direct techniques.

### 2.4. Registration and Ethical Considerations

The research protocol for this clinical trial was thoroughly reviewed and received clearance from the Ethics Commission of the Military Hospital of Sibiu, ensuring that all procedures adhered to ethical guidelines.

All patients participating in the study provided informed consent, fully under-standing the nature of the procedures and their involvement in the research. They also committed to attending all scheduled follow-up appointments over the course of the two-year monitoring period.

Additionally, the research protocol was registered with the Open Science Framework (OSF) under the registration code osf.io/tvn8r, ensuring transparency and adherence to open scientific practices. By registering the study with OSF, the researchers aimed to make the methodology, objectives, and outcomes accessible for replication and scrutiny by the broader scientific community, while still maintaining the necessary confidentiality measures due to the sensitive nature of the participants involved.

### 2.5. Stamp Technique

Out of the 147 direct composite restorations performed, 93 were completed using the stamp technique, as it was deemed the most appropriate for those specific clinical situations. Consequently, the subsequent section will detail the procedural steps of the stamp technique, given its predominance in the study sample. The objective of this investigation is not to provide a case-based description of all direct techniques, but rather to evaluate and compare the performance of direct versus semi-direct techniques over time. Therefore, the authors considered it unnecessary to present the full clinical protocol for each direct technique, as these procedures are already well-documented and widely studied in the literature.

**Figure 1:**
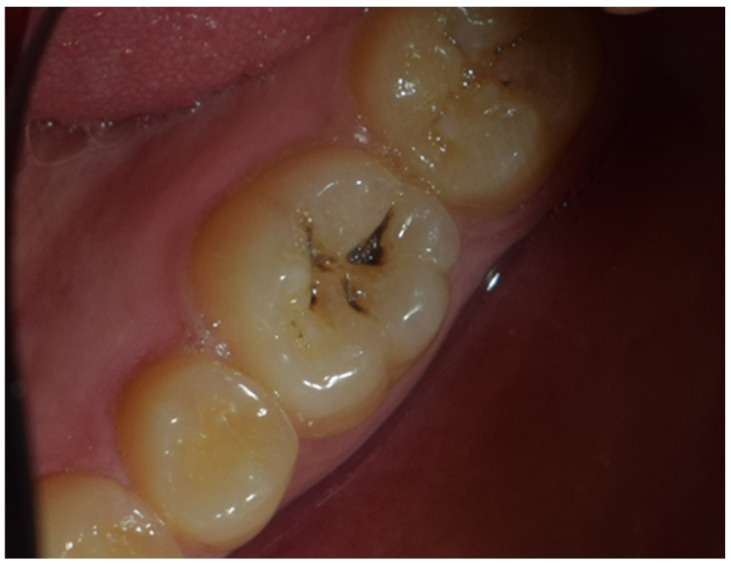
Initial aspect of tooth 36 with Class I cavity.

**Figure 2:**
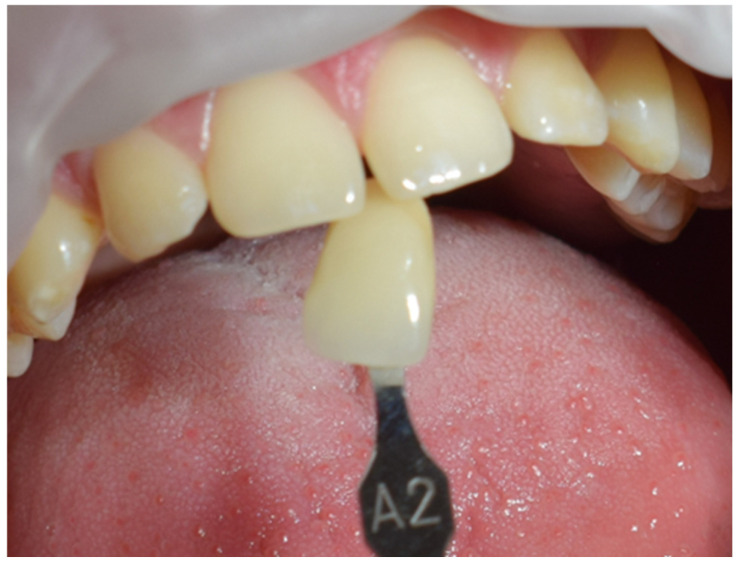
Shade selection using the Vita shade guide.

**Figure 3:**
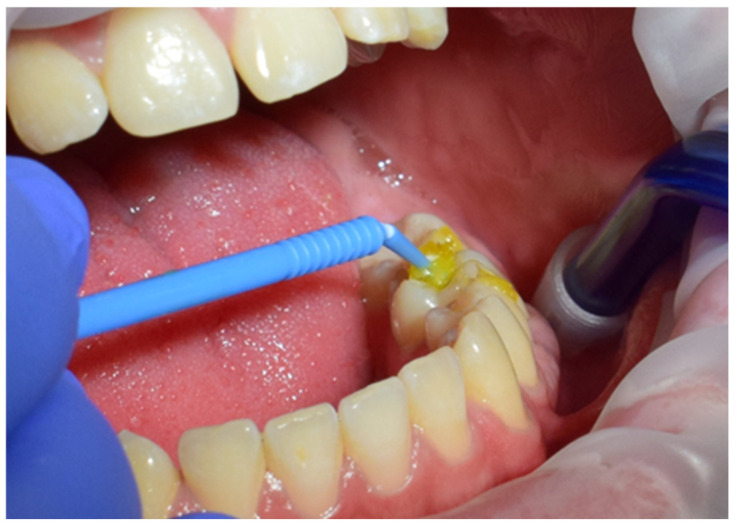
Application of Vaseline to prevent stamp adhesion.

**Figure 4:**
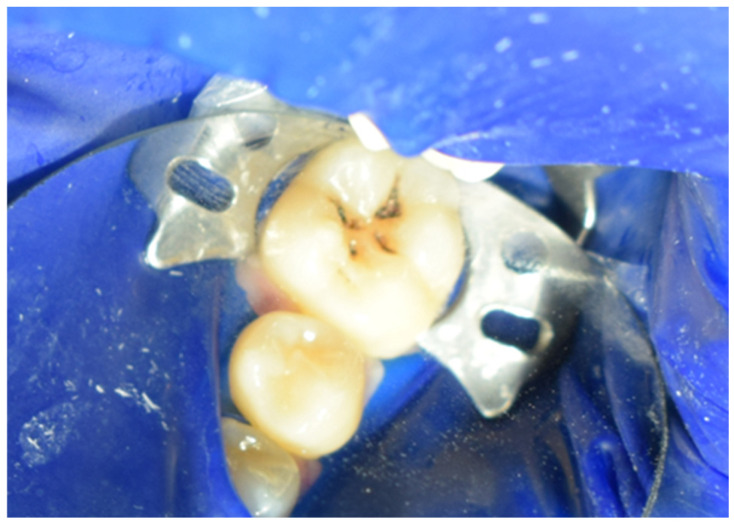
Isolation of the operative field with a rubber dam.

The restoration process using the stamp technique on tooth 36 began with the initial assessment of a Class I cavity (Figure 1). Colour selection was performed using a Vita shade guide to ensure accurate matching of the restorative material to the natural tooth shade (Figure 2). Before proceeding, Vaseline was applied to the tooth surface to prevent the stamp material from adhering (Figure 3), followed by isolation of the operative field using a rubber dam for moisture control and contamination prevention (Figure 4).

**Figure 5:**
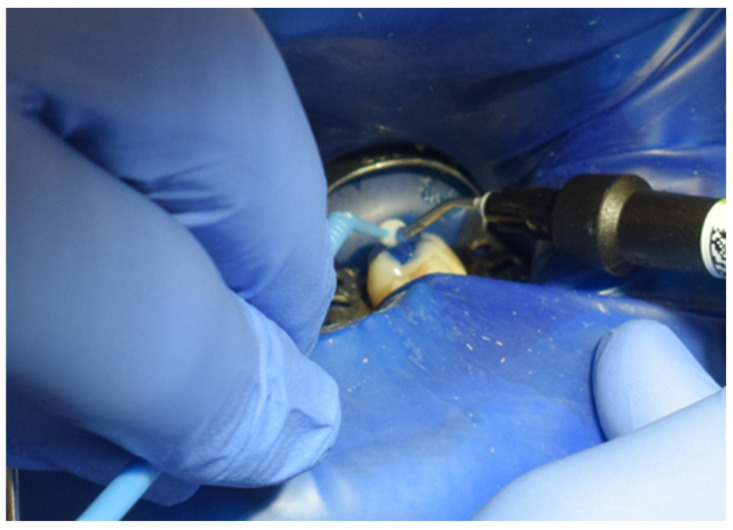
Attachment of micro-applicator handle to the stamp.

**Figure 6:**
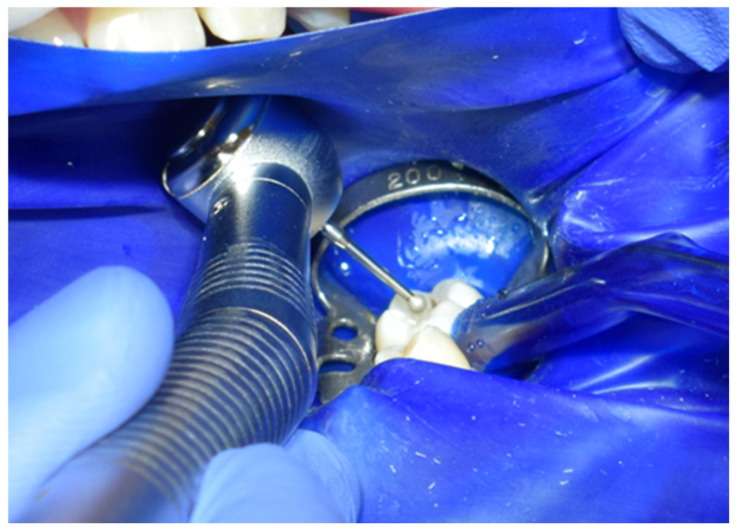
Preparation of cavity using diamond and tungsten carbide burs.

**Figure 7:**
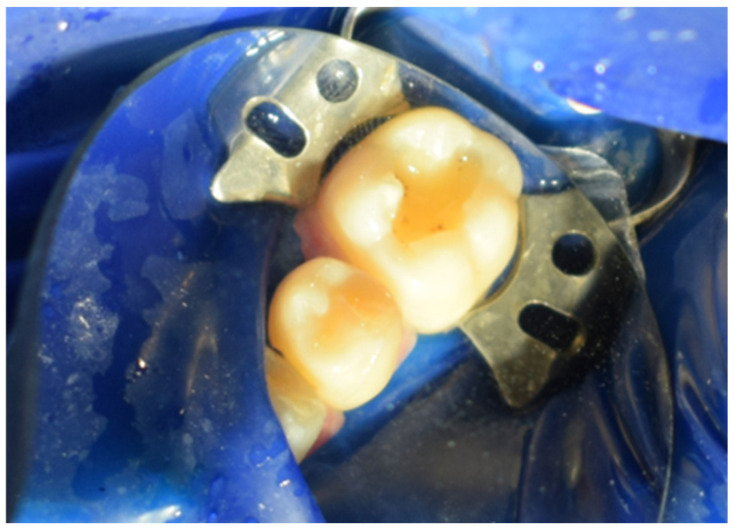
Aspect of the cavity after preparation.

Subsequently, the stamp itself was created using fluid dam material, precisely adapting it over the occlusal anatomy of the tooth. A micro-applicator was attached as a handle to facilitate manipulation and precise placement of the stamp later in the procedure (Figure 5). Tooth preparation was then performed, initially utilising a globular diamond bur mounted on a high-speed handpiece for enamel penetration, followed by a tungsten carbide bur at conventional speed to remove any soft, affected dentin (Figure 6). The resulting cavity exhibited clean and clearly defined margins, ready for restorative materials (Figure 7).

**Figure 8:**
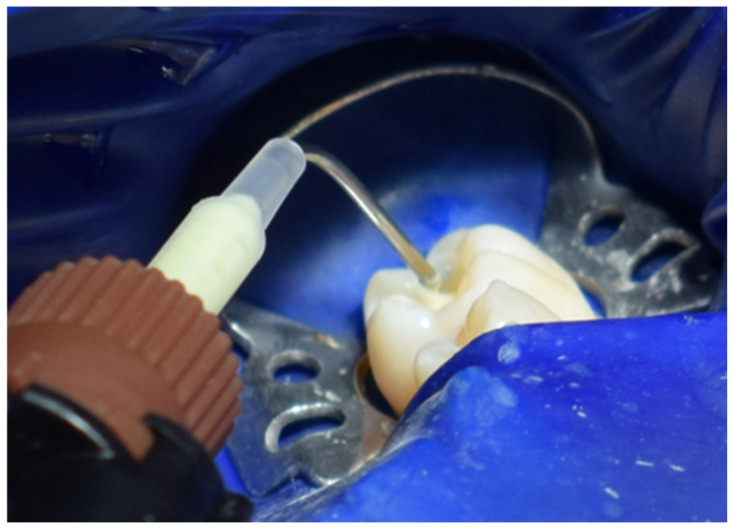
Application of Calcimol LC (VOCO) as cavity base isolation.

**Figure 9:**
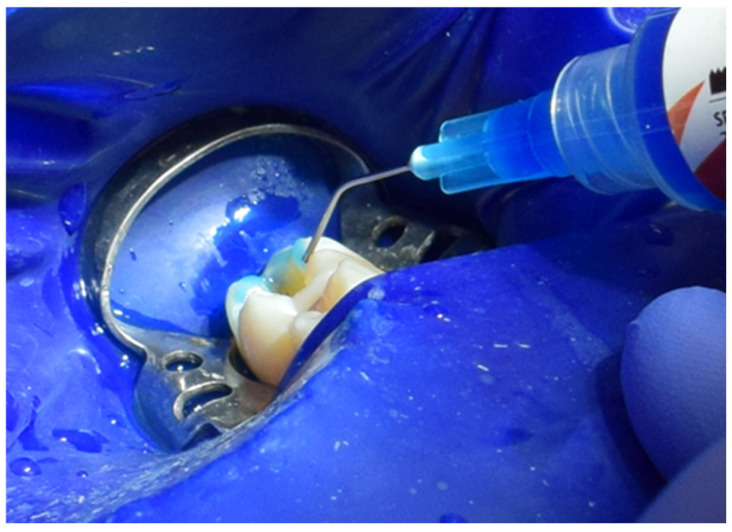
Enamel etching using Blue Etch (Cerkamed).

**Figure 10:**
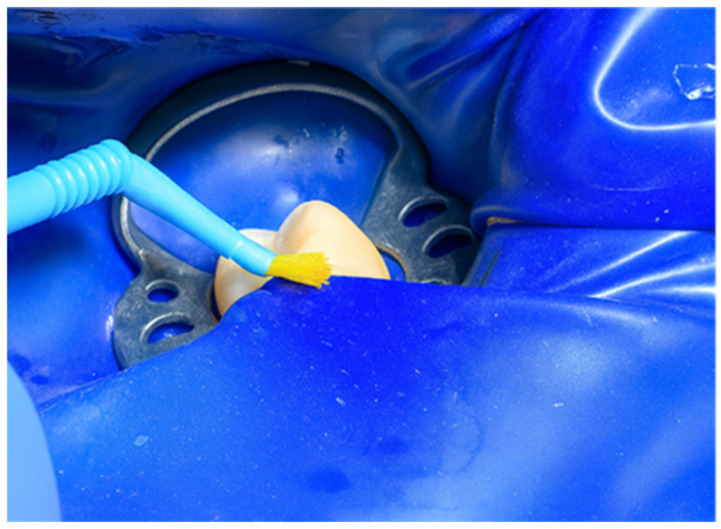
Application and polymerization of G-Premio Bond adhesive (GC).

Calcimol LC by VOCO (Cuxhaven, Germany) was then applied to protect and isolate the base of the cavity (Figure 8). Enamel was etched for 40 s using Blue Etch by Cerkamed (Stalowa Wola, Poland), creating an optimal matte surface for adhesive bonding (Figure 9). A universal adhesive, G-Premio Bond by GC, was then carefully applied and polymerized for 20 s, establishing a robust bond between the tooth structure and restorative materials (Figure 10).

**Figure 11:**
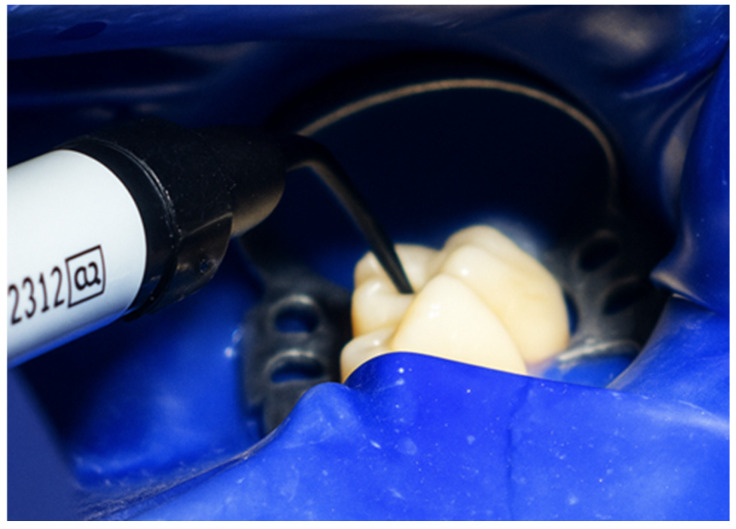
Placement of G-ænial Universal Flo (GC) at cavity base.

**Figure 12:**
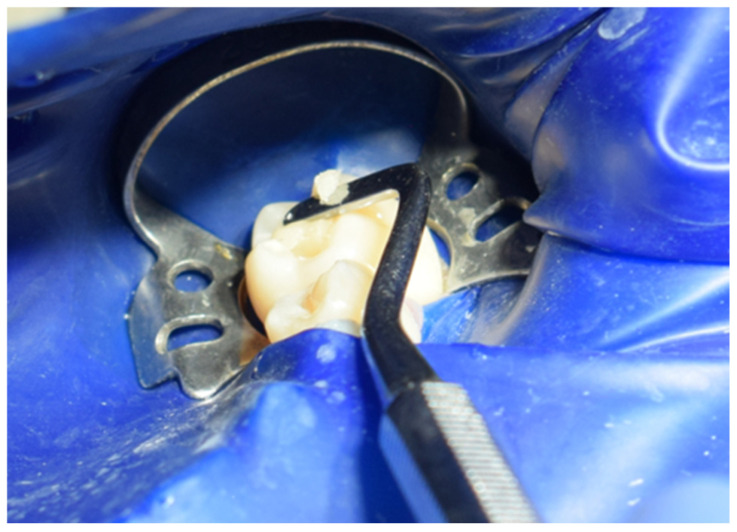
Incremental filling of the cavity with G-ænial composite (GC).

**Figure 13:**
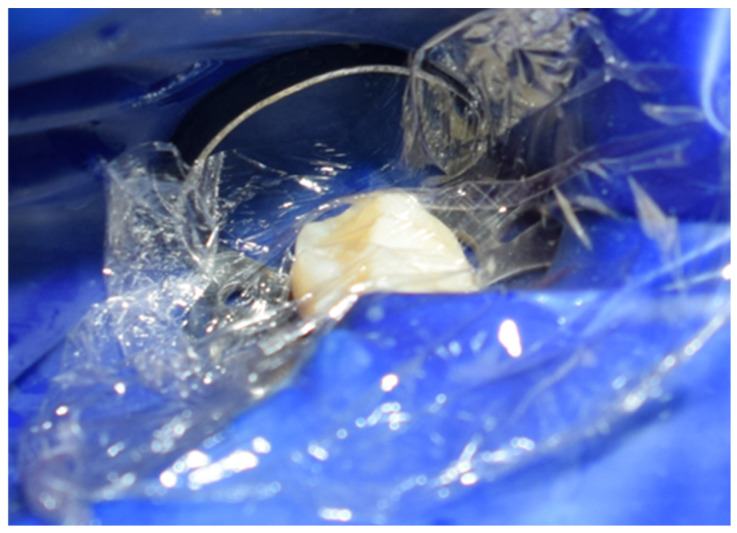
Placement of food-grade plastic film over the composite material.

**Figure 14:**
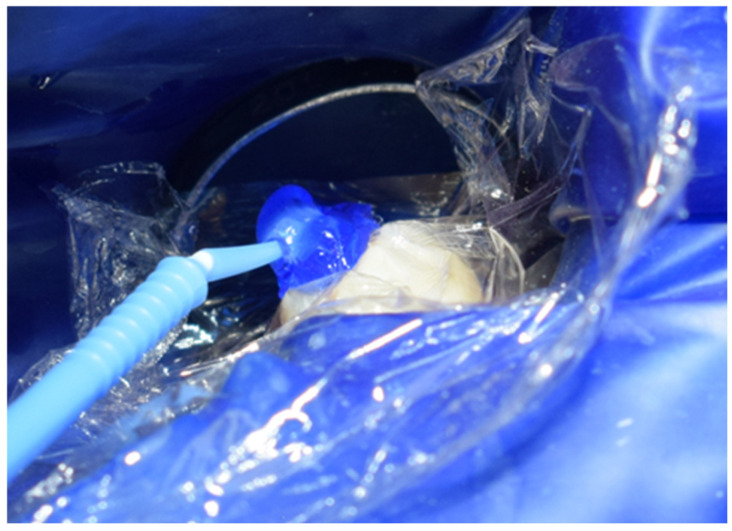
Positioning and adaptation of stamp onto the composite.

The restorative phase commenced with a thin layer of composite flow G-ænial Universal Flo by GC at the cavity base, ensuring adequate material adaptation, minimising polymerization shrinkage, and emulating the semi-direct restoration technique presented forward (Figure 11). Subsequently, G-ænial composite by GC was incrementally placed to fill the cavity completely (Figure 12). A layer of food-grade plastic film was positioned over the composite to prevent adhesion of the matrix to the material (Figure 13). This was then gently seated on the occlusal surface, excess composite material was removed while maintaining its stability, and the anatomical accuracy was verified (Figure 14).

**Figure 15:**
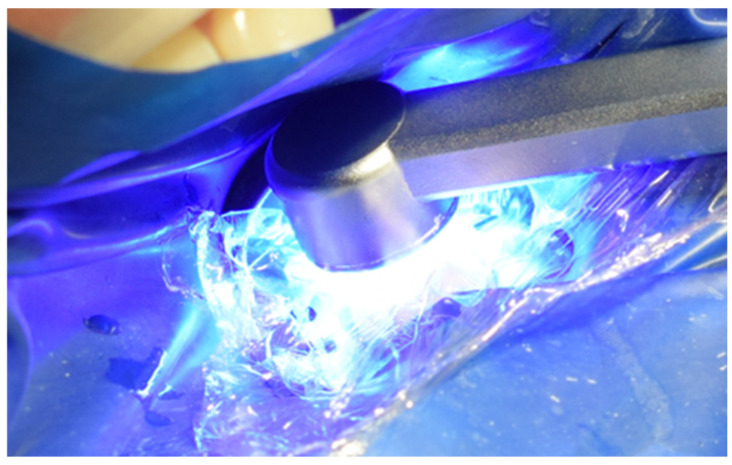
Appearance after stamp removal and photopolymerization.

**Figure 16:**
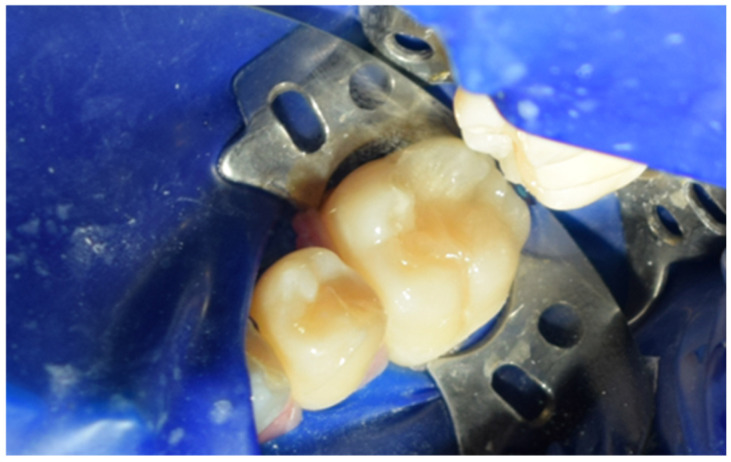
Immediate aspect after removing protective plastic film.

**Figure 17:**
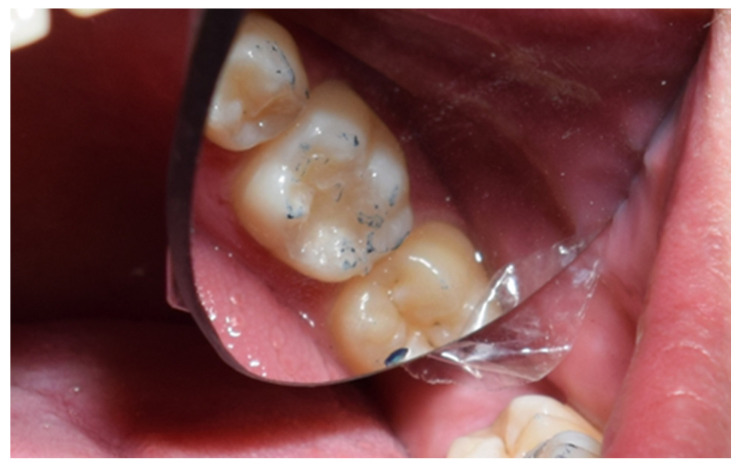
Occlusion check performed articulating paper.

**Figure 18:**
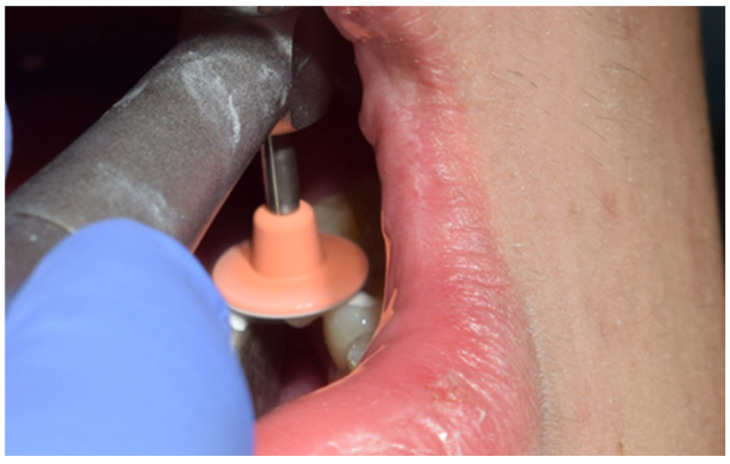
Polishing and smoothing of restoration margins.

**Figure 19:**
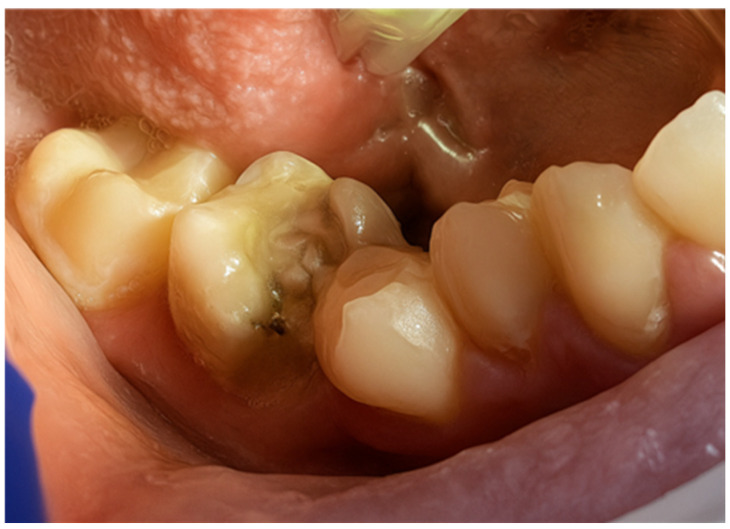
Initial aspect of the tooth showing the cavity field using a rubber.

After careful removal of the stamp, the composite was photopolymerized for 35 s to ensure complete curing and structural integrity (Figure 15). The immediate post-curing appearance demonstrated accurate anatomical restoration upon removing the protective film (Figure 16). Occlusion was carefully verified, first by patient feedback and then confirmed with articulating paper adjustments (Figure 17). Finally, polishing was performed using abrasive paste and a rubber polishing instrument to achieve smooth margins and a highly lustrous finish (Figure 18). The completed restoration demonstrated aesthetic and functional integration with the natural dentition (Figure 19).

### 2.6. Semi-Direct Onlay Technique

**Figure 20:**
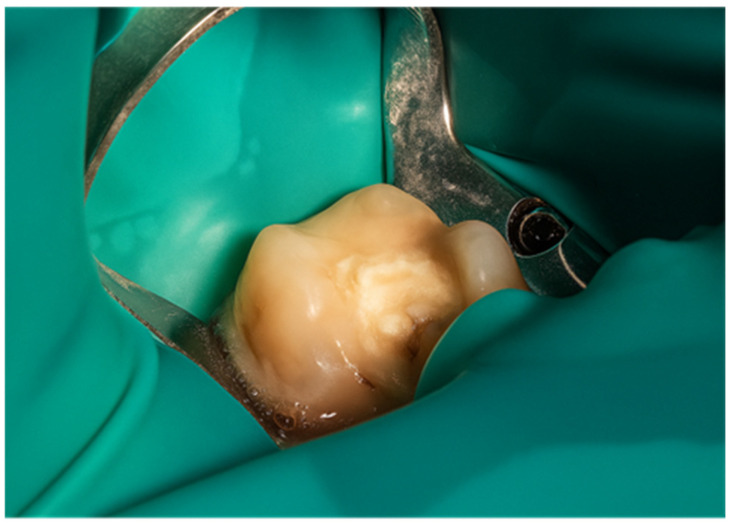
Isolation of the operative dam.

The semi-direct onlay restoration was selected in this case due to the extent of structural loss, which exceeded the indications for a direct composite restoration and necessitated cuspal coverage to ensure long-term stability and functional integrity. The clinical procedure began with the assessment of tooth 4.7, where an extensive cavity was identified, requiring a restorative intervention (Figure 19). After proper isolation using a rubber dam to maintain a dry and contamination-free environment (Figure 20), cavity preparation commenced.

**Figure 21:**
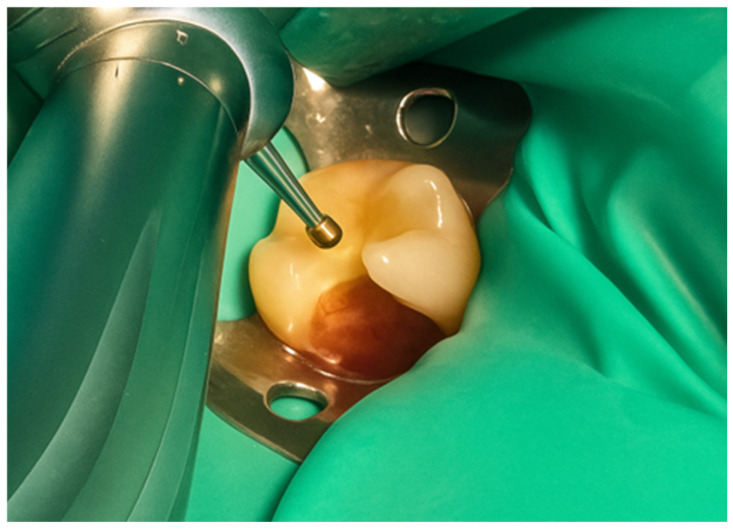
Cavity preparation using a diamond bur (high-speed) and a tungsten carbide bur (slow-speed) to remove affected dentine.

**Figure 22:**
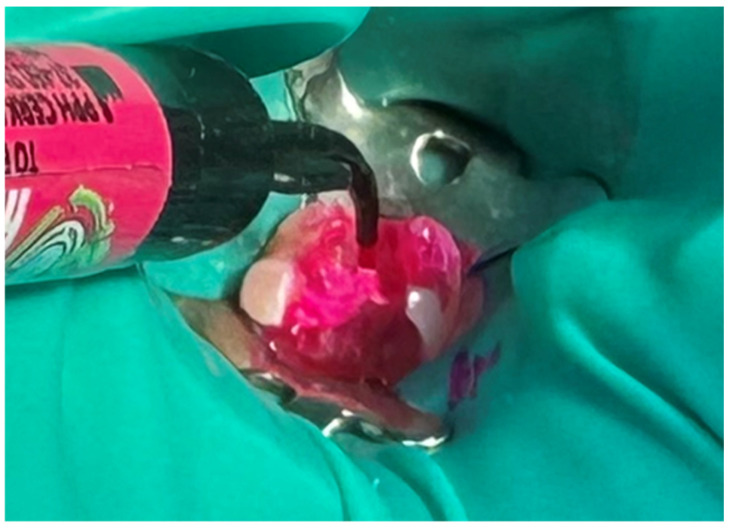
Application of Red Detector (Cerkamed) to check for remaining carious tissue.

**Figure 23:**
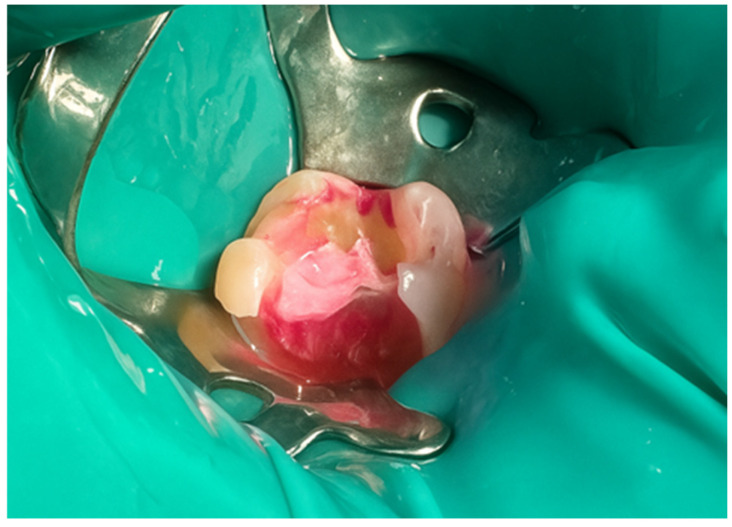
Appearance after rinsing off the caries indicator.

**Figure 24:**
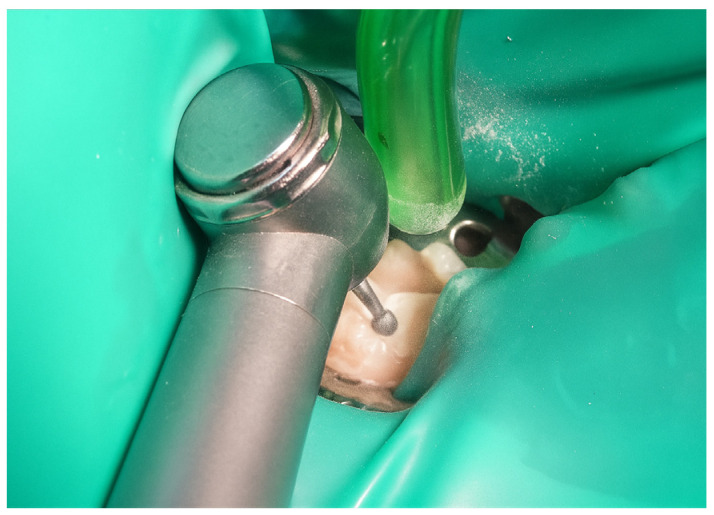
Aspect of the cavity after complete removal of damaged tissue.

First, a diamond bur attached to a high-speed handpiece was employed to outline the cavity margins, followed by a tungsten carbide bur at low speed to meticulously remove affected dentine, ensuring a clean and stable cavity base (Figure 21). Due to the large cavity size, Red Detector (Cerkamed) was utilised to verify the complete removal of carious tissues, confirmed visually after rinsing (Figure 22 and Figure 23). The coloured tissue was then removed (Figure 24).

**Figure 25:**
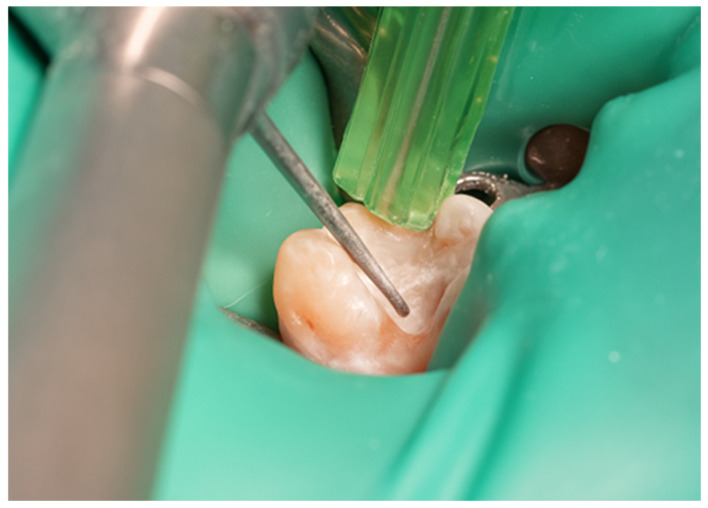
Divergent preparation of cavity walls using a prepared tooth with gin-cylindrical diamond bur.

**Figure 26:**
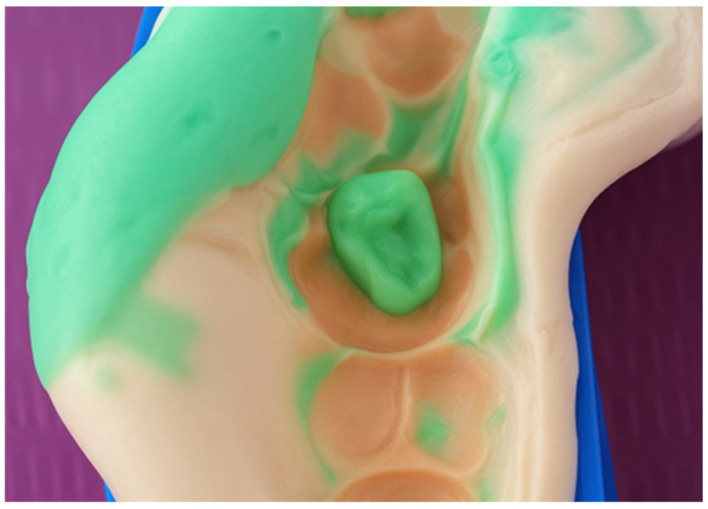
Aspect of the detailed impression obtained.

**Figure 27:**
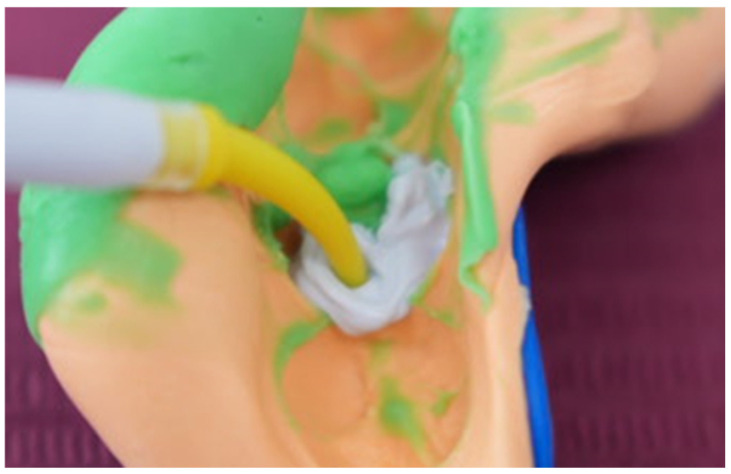
Creation of a rapid dental cast using Occlufast Rock (Zhermack) to avoid waiting time.

Following this, the cavity walls were refined and prepared divergently using a cylindrical diamond bur, optimising insertion for the onlay restoration (Figure 25). The impression procedure was carried out using President Putty Super Soft and President Regular Body (Coltene, Altstätten, Switzerland), capturing accurate details of the cavity preparation (Figure 26). To expedite the process, an immediate replica of the tooth was produced chairside using Occlufast Rock (Zhermack, Badia Polesine, Italy), applying a layer of Vaseline to prevent adherence between the cast and the impression (Figure 27), instead of the classic stone cast, which would require hours to dry or even a scanned and printed model, which would also require at least a couple of hours and additional equipment.

The onlay fabrication started with the application of a thin layer of G-ænial Universal Flow composite (GC) to ensure adaptation to intricate cavity details, followed by polymerization. The anatomical restoration was systematically performed on the tooth replica using G-ænial composite (GC), first reconstructing the lingual cusps, then the buccal wall, and finally the buccal cusps with a more viscous composite, meticulously shaping the natural morphology. Upon completion, the finished onlay was removed from the cast and carefully evaluated.

**Figure 28:**
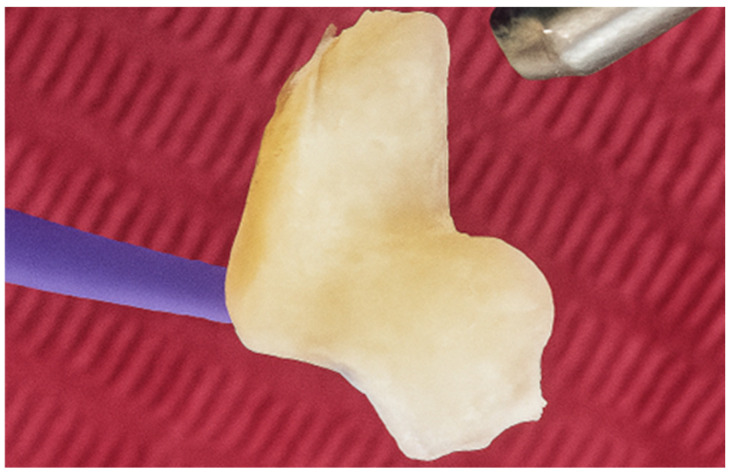
Internal surface sandblasted with 110 µm Al_2_O_3_.

**Figure 29:**
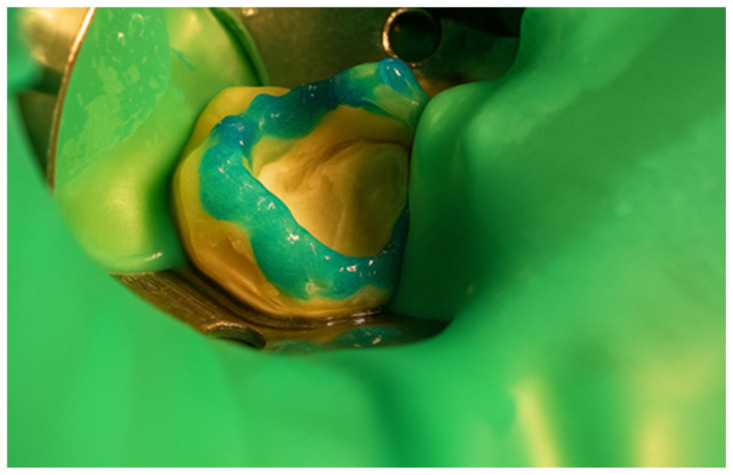
Enamel etching using Blue Etch (Cerkamed).

To enhance bonding, the internal surface of the onlay was sandblasted with 110 µm aluminium oxide particles, preparing it for adhesive cementation (Figure 28). After verifying the intraoral occlusal marginal adaptation and the fit of the onlay, adhesive cementation was performed. A concentrated solution of chlorhexidine (Consepsis, Ultradent, South Jordan, UT, USA) was first applied to the tooth surface, followed by selective enamel etching with 37% phosphoric acid for 10 s (Figure 29). The etchant was thoroughly rinsed with water until no visible residues remained, after which Consepsis was reapplied and gently air-dried.

**Figure 30:**
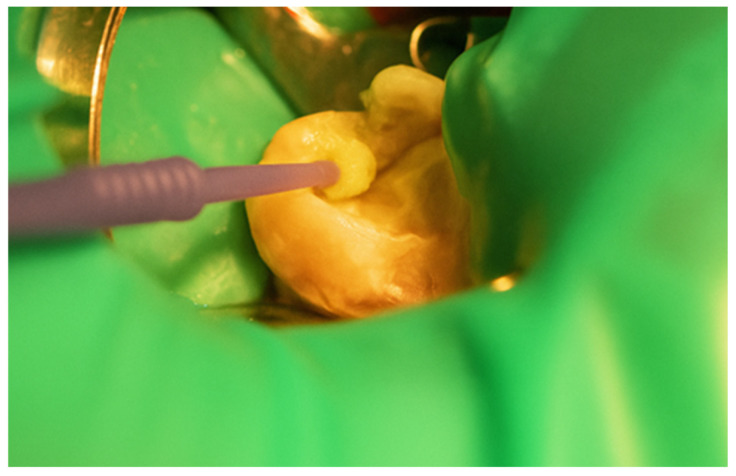
Application of Multilink Primer A/B (Ivoclar).

**Figure 31:**
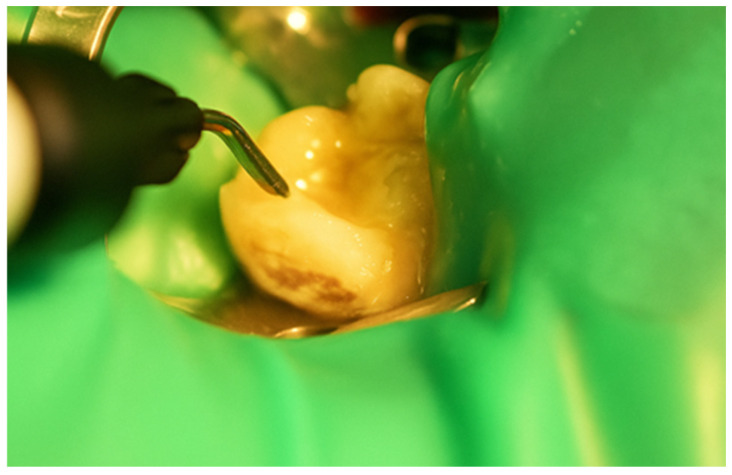
Placement of Multilink (Ivoclar) layer in the cavity as adhesive medium.

**Figure 32:**
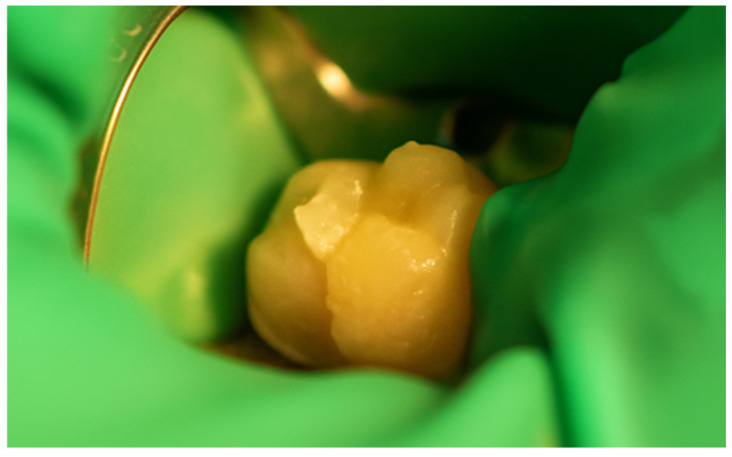
Seating of the onlay.

**Figure 33:**
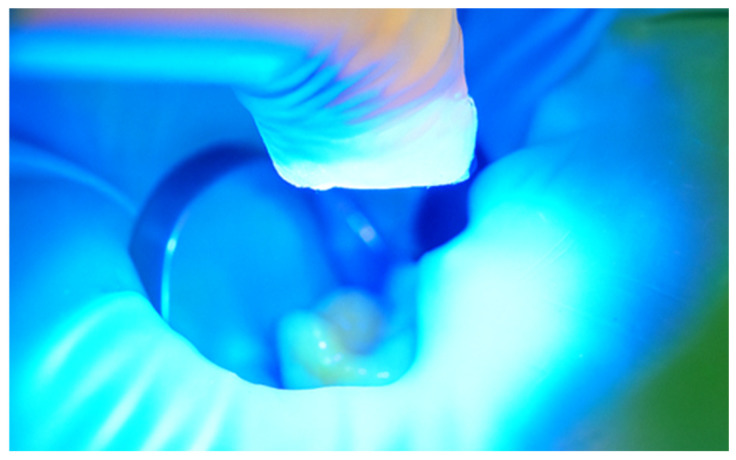
Photopolymerization of the restoration under maintained pressure for 35 s.

**Figure 34:**
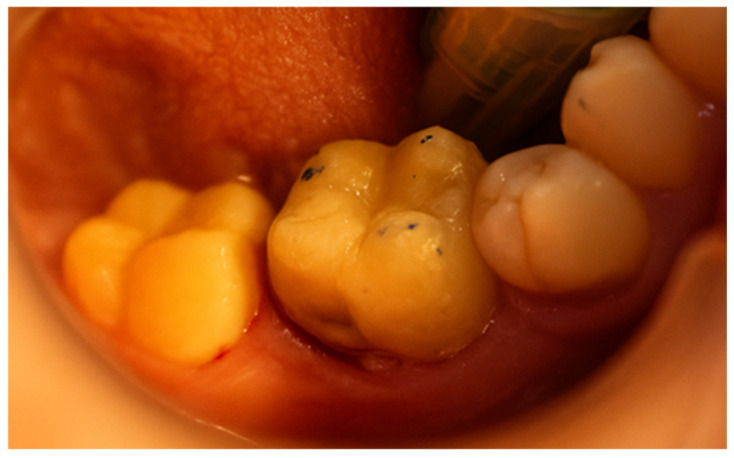
Occlusion verification using articulating paper.

**Figure 35:**
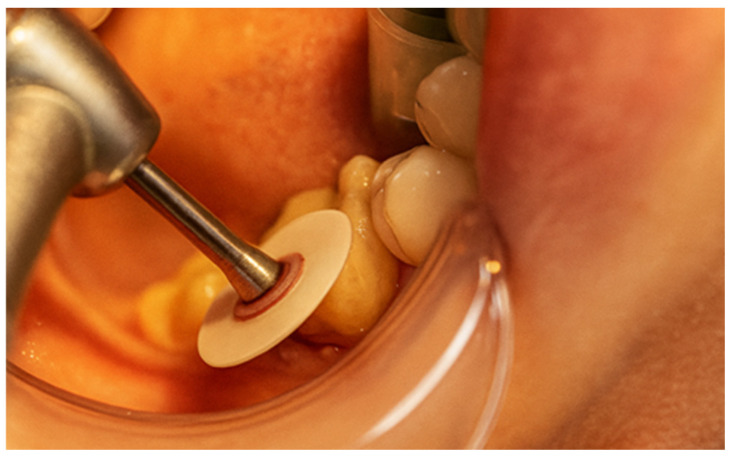
Polishing of the restoration surface.

Subsequently, Multilink Primer A/B from Ivoclar was applied to the prepared surface with a microbrush for 30 s and gently air-dried (Figure 30). Multilink Automix (Ivoclar, Schaan, Liechtenstein) resin cement was dispensed directly onto the internal surface of the cavity, which was then seated into the cavity (Figure 31). After placement(Figure 32), each surface was light-cured for 1–3 s to achieve a gel state, and the excess cement was carefully removed with a scaler. The restoration margins were covered with glycerin gel (air blocker) and polymerized for an additional 20 s (Figure 33). Finally, the occlusion was re-evaluated (Figure 34), and the restoration margins were polished to ensure optimal adaptation and surface finish (Figure 35).

### 2.7. Aesthetic Evaluation

The aesthetic evaluation of composite restorations is a critical component of overall patient satisfaction. In this study, aesthetics were assessed at four different time points: immediately after the restoration (T0), at 6 months (T1), 1 year (T2), and 2 years (T3). At T0, the initial appearance of the restoration was rated immediately after the finishing and polishing procedure. Over time (T1, T2, and T3), the aesthetic outcome was reassessed during follow-up visits to check for discoloration, surface dullness, and any other aesthetic changes that might have occurred. Both patient feedback and clinical examination were used to track the aesthetic performance of each restoration.

Patients’ satisfaction with the visual appearance of their restorations was measured on a scale from 1 to 5, where 1 indicated dissatisfaction and 5 represented maximum satisfaction. Factors considered in the aesthetic evaluation included colour match with the adjacent teeth, the natural appearance of the restoration’s translucency, and how well the restoration blended with the natural contours of the tooth. In this scale: (1) Poor—the restoration is visibly different in shade or opacity, with obvious demarcations; (2) Fair—moderate mismatch in colour or translucency and the restoration blends somewhat but is still noticeable, with visible transitions between the restoration and natural tooth structure; (3) Good—the restoration blends well with adjacent teeth, though slight differences in shade or texture may be noticeable; (4) Very Good—the restoration is nearly indistinguishable from the surrounding teeth in normal viewing conditions, with only minimal differences under close examination and (5) Excellent—the restoration is completely indistinguishable from adjacent teeth in terms of colour, translucency, and overall appearance, the patient is highly satisfied with the aesthetic outcome.

### 2.8. Surface Finish Evaluation

The surface finish of composite restorations plays a crucial role in both aesthetics and longevity, influencing plaque accumulation and overall durability. To evaluate the surface finish, clinical examinations were conducted at T0, T1, T2, and T3. At T0, surface smoothness was assessed immediately after polishing using tactile feedback and magnification tools. A smooth and glossy surface is essential for reducing bacterial plaque retention, which in turn helps prevent recurrent decay and maintains aesthetic quality. Over time, the surface finish was evaluated for signs of roughness, wear, and potential microcracks. Instruments like tactile feedback and clinical observations under magnification were used to objectively measure the texture of the surface.

Restoration surfaces were rated based on the degree of roughness or smoothness, with a scale ranging from rough (1) to perfectly smooth (5). Rough (1): The surface is very rough to the touch, with visible irregularities. Over time the restoration shows significant wear and attracts plaque easily. High likelihood of plaque buildup and recurrent caries. Slightly Rough (2): The surface has noticeable roughness but is acceptable. Some plaque retention may occur, and the surface shows early signs of wear or cracking. Smooth (3): The surface is relatively smooth, with minimal rough spots. Plaque retention is limited, and the surface appears uniform. No signs of wear or cracking yet. Very Smooth (4): The surface is smooth and polished, with no noticeable irregularities. Plaque retention is minimal, and the surface finish remains intact over time. Perfectly Smooth (5): The surface is exceptionally smooth and glossy, with no visible imperfections. It resists plaque buildup and maintains its finish over time without any visible degradation.

### 2.9. Wear Resistance Evaluation

Wear resistance is a vital functional aspect of posterior composite restorations due to the significant occlusal forces exerted in this region. Wear was evaluated at T1, T2, and T3, using both clinical observation and quantitative measurements such as the amount of material lost over time. The wear evaluation included assessing the reduction in height and volume of the composite material, especially in areas under heavy occlusal load. Any flattening of the occlusal anatomy, loss of cusp definition, or general material degradation were documented to understand how each technique, direct or semidirect onlay, held up over time. This information provided insight into the long-term durability of the materials and techniques used.

The wear resistance of each restoration was rated on a scale from 1 (severe wear) to 5 (no wear). Severe Wear (1): Significant loss of material, with noticeable flattening of occlusal anatomy or cusp definition. Restoration requires immediate repair or replacement. Moderate Wear (2): Visible wear, with moderate loss of height or volume in the restoration. Occlusal anatomy is noticeably compromised, and adjustments may be required. Mild Wear (3): Mild but noticeable wear. Some flattening of the occlusal surface, but the restoration still functions well without the need for immediate adjustment. Minimal Wear (4): Very little wear is visible, with only minor changes in the occlusal anatomy. The restoration remains functional and aesthetic without any adjustments. No Wear (5): No detectable wear. The restoration remains in its original form, with perfect preservation of occlusal anatomy and functional integrity.

### 2.10. Marginal Integrity and Microleakage Evaluation

Marginal integrity and microleakage were assessed at T1, T2, and T3. Microleakage is a concern because it can lead to secondary caries, sensitivity, and failure of the restoration. During follow-up examinations, marginal integrity was tested by applying a dye solution around the margins of the restorations and then observing the degree of penetration along the tooth-restoration interface under magnification. A well-sealed margin should prevent dye penetration, while any leakage indicates potential gaps that could allow bacterial ingress. Additionally, clinical evaluations were performed to check for visible signs of deterioration at the margins, such as chipping or marginal discoloration.

The marginal integrity was rated on a scale from 1 (severe leakage) to 5 (no leakage). Severe Microleakage (1): Dye penetration through the entire margin of the restoration. Presence of secondary caries, sensitivity and restoration failure. Immediate intervention is required. Moderate Microleakage (2): Significant dye penetration along the margins. Possible presence of caries visible on consult or radiological examinations, sensitivity is present but at a lower intensity, repair may be necessary. Mild Microleakage (3): Mild dye penetration, small gaps in the marginal seal. Secondary caries may be seen on radiological examination and sensitivity is detected, the restoration may still function adequately. Minimal Microleakage (4): Very minimal dye penetration, with a visible intact marginal seal, secondary caries are not present and sensitivity is present at very high intensity of stimuli. The restoration is functionally sound and should not require further intervention. No Microleakage (5): Perfect marginal seal, with no dye penetration. There are no caries or sensitivity. The restoration is securely bonded to the tooth with no risk of leakage or failure.

### 2.11. Time and Efficiency Evaluation

The time and efficiency of each technique were documented by recording the total time spent on each restoration, from preparation to finishing at T0. This included time spent on isolation, cavity preparation, material placement, finishing, and patient comfort evaluations. The efficiency of the technique was assessed by comparing the total procedure times for the direct technique versus the semidirect onlay technique. Shorter procedure times with optimal outcomes were considered more efficient.

The efficiency of the procedure in terms of the total time taken to complete the restoration was evaluated on a scale from 1 to 5 where (1) is Very Inefficient and (5) is Highly Efficient, focusing on both the time spent with the patient’s mouth open and the overall procedure duration. A session exceeding 20 min with the mouth open is considered uncomfortable for the patient, while a total procedure time longer than 40 min is deemed inefficient for the dentist. Very Inefficient (1): The patient spent over 30 min with their mouth open, and the total procedure time exceeded 60 min. Both the patient and dentist experienced significant delays, with the patient exhibiting signs of discomfort. Inefficient (2): The patient spent between 20 and 30 min with their mouth open, and the procedure took 50–60 min in total. The time spent was uncomfortable for the patient, and the procedure was inefficient for the dentist. Moderately Efficient (3): The patient spent approximately 20 min with their mouth open, and the total procedure time ranged from 40 to 50 min. The patient experienced some discomfort, but the procedure was moderately efficient for the dentist. Efficient (4): The patient spent less than 20 min with their mouth open, and the total procedure time ranged from 30 to 40 min. The procedure was efficient for the dentist, with minimal discomfort for the patient. Highly Efficient (5): The patient spent significantly less than 20 min with their mouth open, and the total procedure time was under 30 min. The procedure was highly efficient for the dentist, and the patient was comfortable throughout.

### 2.12. Statistical Analysis

For the statistical analysis of the evaluated criteria, data from the four evaluation time points (T0, T1, T2, and T3) were collected for each criterion (aesthetic, surface finish, wear resistance, marginal integrity, microleakage, time and efficiency). Each criterion was rated on a scale from 1 to 5, and the analysis was aimed at identifying significant differences over time and between the two techniques (direct technique vs. semidirect onlay technique). Descriptive statistics such as means, medians, and standard deviations were first calculated to provide an overall view of the data distribution for each criterion at each time point.

To test for statistically significant differences in the scores over time and between the two techniques, repeated-measures ANOVA was performed [56]. This test was suitable for analysing the repeated measurements (T0 to T3) for the same patients, allowing for the comparison of each criterion’s performance over time. Additionally, paired t-tests or Wilcoxon signed-rank tests were used for pairwise comparisons between time points when appropriate [57]. For the comparison between the two techniques at each time point, independent t-tests or Mann–Whitney U tests were conducted, depending on whether the data followed a normal distribution [58].

All statistical analyses were conducted using SPSS software (Statistical Package for the Social Sciences, version 25) [59] for its robust features in handling repeated measures and non-parametric data. A significance level of *p* < 0.05 was set to determine whether the differences observed were statistically significant. Data visualisation tools within the software were also utilised to create graphs showing the performance trends over time for each criterion.

## 3. Results

**Figure 36:**
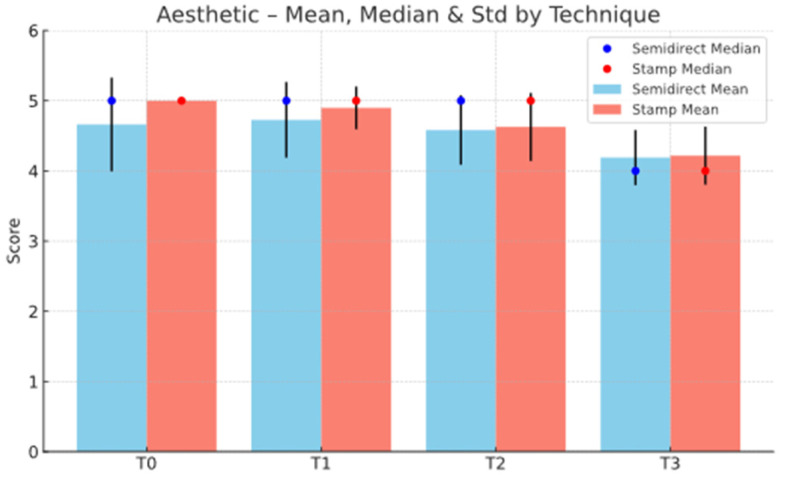
Aesthetic mean, median and std representation.

**Figure 37:**
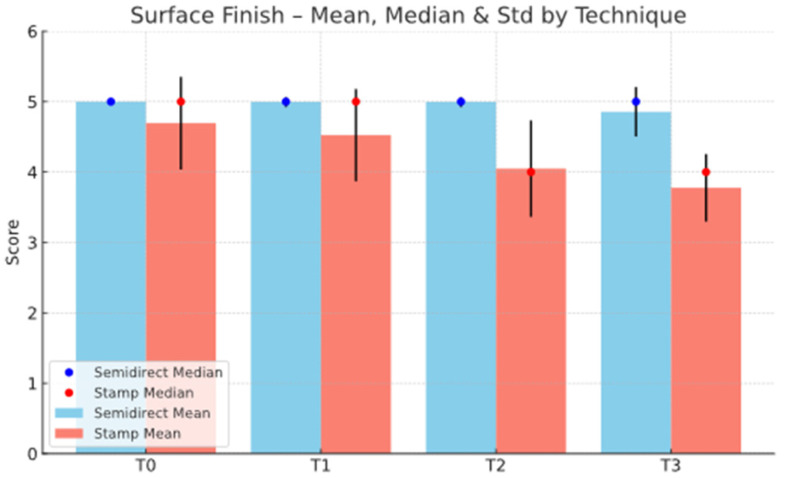
Surface finish mean, median and std representation.

**Figure 38:**
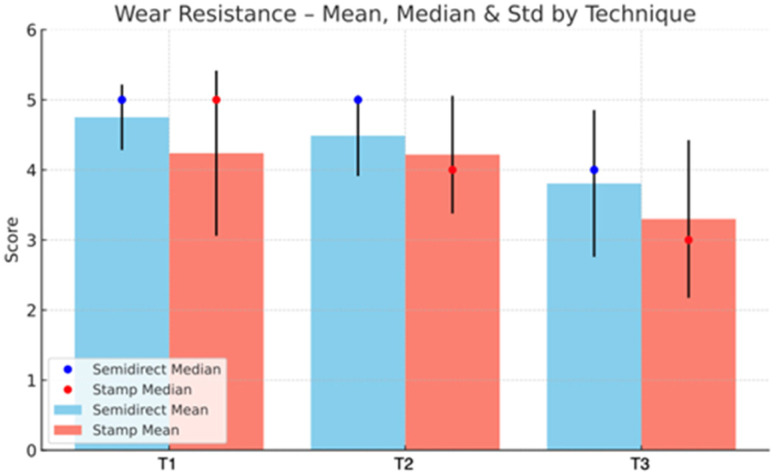
Wear resistance mean, median and std representation.

The statistical analysis highlights clear differences between the Direct and Semidirect Onlay techniques across several evaluated criteria. Table 1 presents a comparative analysis of the two restorative techniques, across multiple clinical parameters over time. The direct technique demonstrates superior early-stage aesthetic performance, with higher mean scores at Aesthetic T0 (5.00 vs. 4.66) and Aesthetic T1 (4.90 vs. 4.73), suggesting better immediate visual outcomes. However, this advantage diminishes by Aesthetic T3 (4.22 vs. 4.19), indicating a slight decline in long-term aesthetic retention for both techniques, as can be seen in Figure 36. In contrast, the semidirect technique exhibits consistently higher values in surface finish at all time points, particularly at T2 (4.99 vs. 4.05) and T3 (4.86 vs. 3.78) as seen in Figure 37, as well as superior wear resistance at T3 (3.81 vs. 3.30), underscoring its superior material durability (Figure 38).

**Figure 39:**
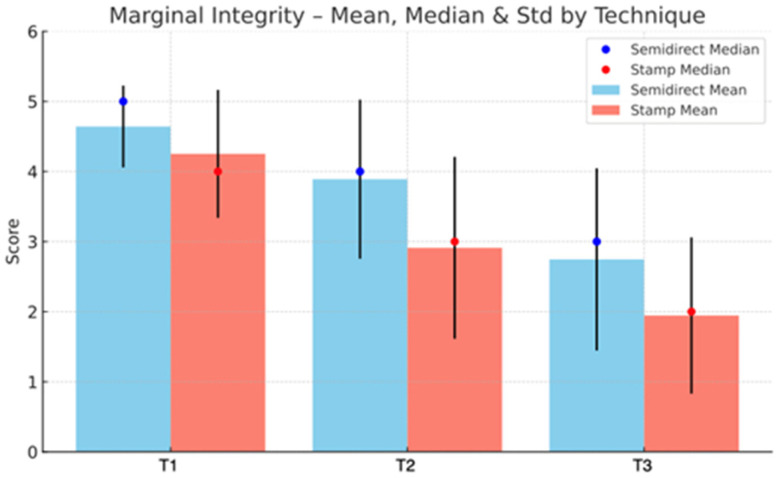
Marginal Integrity mean, median and std representation.

**Figure 40:**
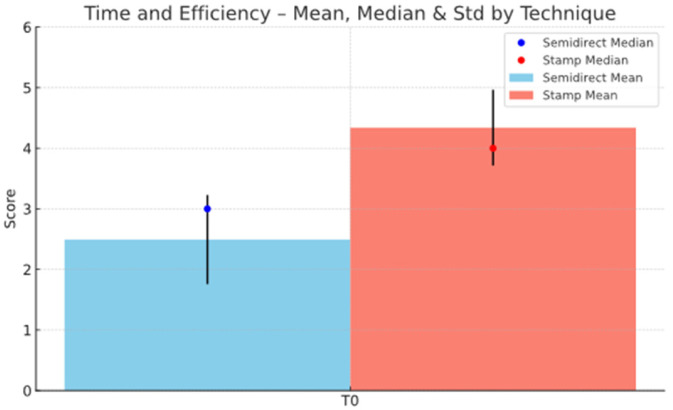
Time and efficiency mean, median and std representation.

Additionally, the semidirect approach outperforms the direct technique in marginal adaptation, especially in later stages; T3 shows a notable difference (2.75 vs. 1.95), highlighting better long-term marginal integrity, as seen in Figure 39. However, the direct technique excels in procedural efficiency, achieving a higher mean score in time and efficiency (4.34 vs. 2.49), reflecting its clinical convenience. This is represented in Figure 40. Overall, while the direct technique is advantageous in aesthetic immediacy and operational speed, the semidirect technique offers superior long-term performance in surface quality, wear resistance, and marginal adaptation, making it more suitable for cases where durability and precision are prioritised.

Repeated-measures ANOVA revealed statistically significant differences over time for all evaluated clinical parameters: aesthetic, surface finish, wear resistance, and marginal integrity (*p* < 0.0001 for all). The results are presented in Table 2. The analysis showed a strong time effect for each domain, with the highest F-value observed in marginal integrity (F(2.694) = 642.79), followed by aesthetic (F(3.1041) = 180.30), surface finish (F(3.1041) = 163.66), and wear resistance (F(2.694) = 137.93). In the context of this clinical study comparing the direct and semidirect onlay techniques for posterior composite restorations, the results of the ANOVA underscore the significant influence of time on the clinical performance of all evaluated parameters: aesthetic outcomes, surface finish, wear resistance, and marginal integrity (all *p* < 0.0001).

**Figure 41:**
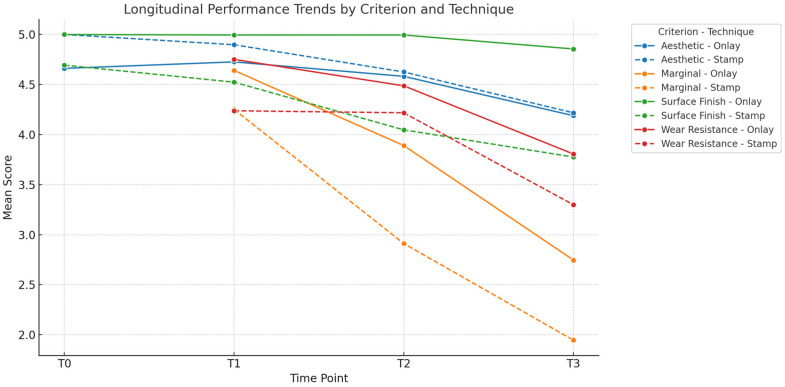
Longitudinal performance trends showing aesthetic and surface finish start high and gradually decline. Wear resistance and marginal integrity show more pronounced degradation over time.

**Figure 42:**
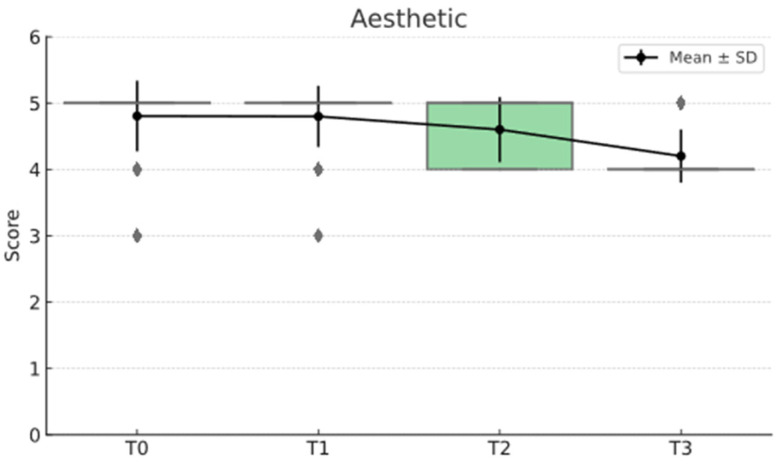
Aesthetics mean and std distribution over time.

**Figure 43:**
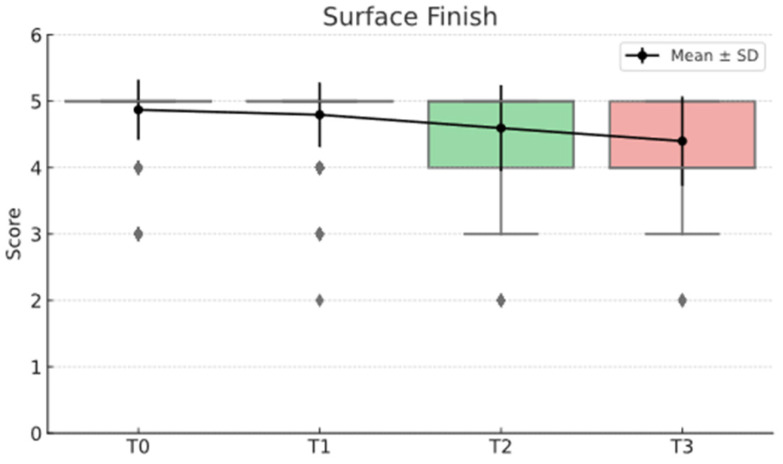
Surface finish mean and std distribution over time.

**Figure 44:**
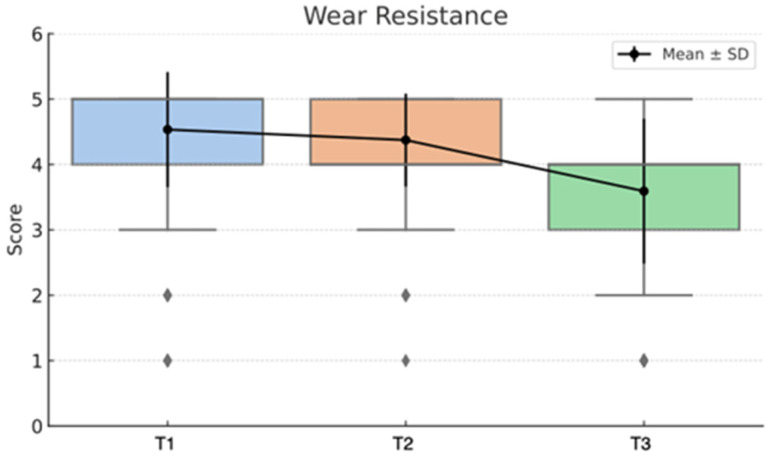
Wear resistance mean and std distribution over time.

**Figure 45:**
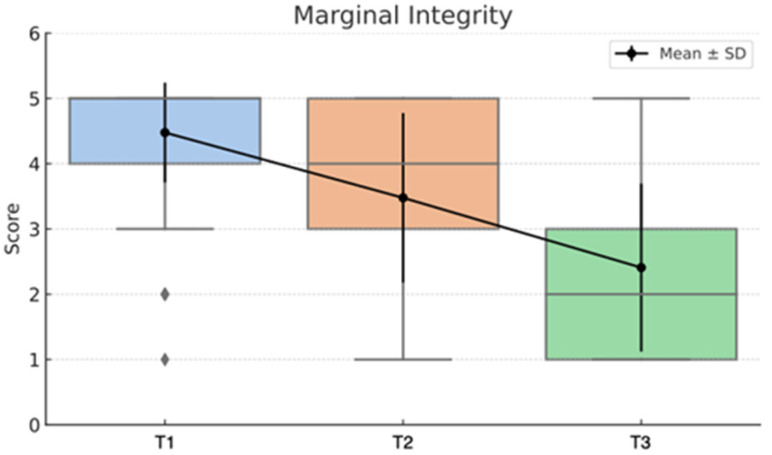
Marginal integ. mean and std distribution over time.

These findings validate the study’s design, which incorporated longitudinal follow-ups at multiple intervals (T0–T3), and highlight that neither technique delivers static outcomes over time. These trends are represented in Figure 41, Figure 42, Figure 43, Figure 44 and Figure 45. The statistically significant time effect confirms that material behaviour and restoration integrity evolve across follow-up periods, reinforcing the importance of temporal evaluation in assessing restorative success. Importantly, this time-dependent variation supports the observed clinical trend in the study: while the direct technique excels in initial aesthetics and procedural efficiency, the semidirect technique provides superior long-term durability, particularly in wear resistance and marginal adaptation. This statistical evidence strengthens the study’s conclusion that the choice of technique should be guided by case-specific priorities, such as swiftly performance and appearance versus long-term structural performance.

**Figure 46:**
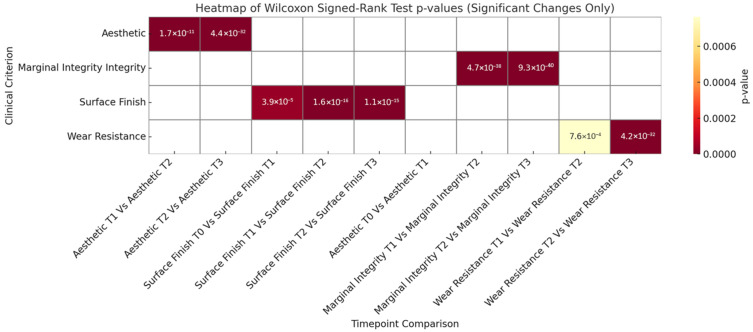
Significant changes over time in clinical criteria: Wilcoxon Signed-Rank Test Results.

The Wilcoxon signed-rank test results revealed statistically significant changes between timepoints for all evaluated clinical criteria, reinforcing the time-dependent nature of restoration performance. Within the aesthetic domain, significant differences were observed between T1–T2 (*p* < 0.0001) and T2–T3 (*p* ≈ 4.4 × 10^−32^), while no significant difference was found between T0 and T1 (*p* = 0.955). This indicates that the immediate aesthetic outcomes of both techniques remained stable during the first six months post-restoration, aligning with the high patient satisfaction observed at T0. These results can be seen in Table 3 and Figure 46. However, noticeable aesthetic degradation began to occur between 6 months and 1 year, becoming even more pronounced by the 2-year follow-up, consistent with the reported discoloration and surface dullness in some direct restorations over time.

For surface finish, all time intervals showed statistically significant changes, with the most dramatic decline occurring between T1 and T2 (*p* ≈ 1.6 × 10^−16^) and between T2 and T3 (*p* ≈ 1.1 × 10^−15^), as seen in Table 3. This supports the study’s clinical observations that restorations, particularly those placed using the direct techniques, begin to lose surface smoothness after the initial post-operative period. Likewise, wear resistance and marginal integrity exhibited highly significant changes between follow-up periods, especially between T2 and T3 (*p* < 10^−32^ and *p* < 10^−38^, respectively). These findings confirm that material degradation and microleakage become critical concerns after one year, particularly in restorations subjected to high occlusal stress or insufficient marginal sealing.

In the context of this study comparing the direct and semidirect techniques, these results emphasise the importance of long-term evaluation. While initial performance may appear similar, particularly in aesthetics, the semidirect technique demonstrates superior durability over time in surface finish, wear resistance, and marginal adaptation. The statistical data supports the clinical conclusion that although the direct technique offers time efficiency and immediate visual appeal, it is more prone to functional decline beyond the first year. Therefore, for patients requiring long-lasting posterior restorations, especially under high occlusal loads, the semidirect technique remains the more predictable and resilient option.

**Figure 47:**
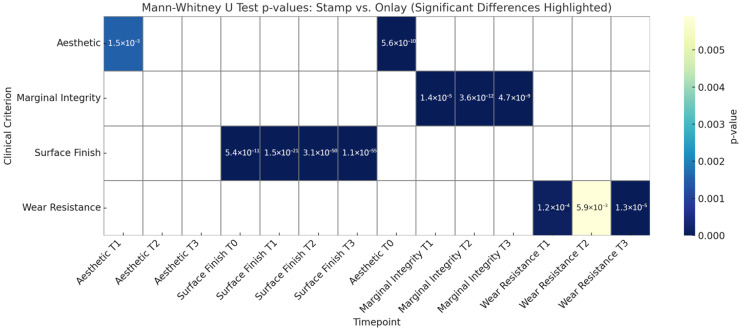
Significant differences between direct and onlay techniques across clinical criteria (Mann–Whitney U Test).

The Mann–Whitney U test results provide a non-parametric comparison of clinical scores between the semidirect (onlay) and direct techniques across all evaluated timepoints and criteria. Statistically significant differences were found primarily in the early stages of treatment, as can be seen in Table 4 and Figure 47. For example, at Aesthetic T0, the semidirect and direct techniques demonstrated a significant difference in aesthetic ratings (U = 11,392.5, *p* < 0.0001), with the direct technique achieving higher scores. This aligns with the clinical advantage of the direct technique in producing immediate high-fidelity occlusal anatomy and superior initial visual appeal. A significant difference remained at T1 (*p* = 0.0015), but by T2 and T3, the difference was no longer significant (*p* > 0.4), indicating that the initial aesthetic advantage of the direct technique diminishes over time, possibly due to wear, discoloration, or marginal degradation.

A similar trend is evident in the Surface Finish category. At T0, the Mann–Whitney U test revealed a highly significant difference (U = 17,688.0, *p* < 0.0001), once again favouring the direct technique. However, as time progresses, the surface quality of restorations placed with the direct method declines, eventually showing no statistically significant difference at later timepoints. This supports clinical observations from the study that the direct technique excels in immediate post-operative smoothness but does not maintain that advantage long-term. The semidirect technique, although initially slightly less refined in finish, maintains better surface characteristics over time, likely due to its controlled lab-based polymerization and finishing.

When evaluating wear resistance and marginal integrity, the Mann–Whitney tests reveal a reverse trend: no significant differences at early timepoints, but potential differences emerging as time progresses. These findings suggest that while both techniques are comparable in functional stability shortly after placement, the semidirect technique begins to outperform the direct technique in functional resilience as the restoration ages. This aligns with the broader findings of this study, which showed greater long-term durability and marginal adaptation in the semidirect group.

Overall, the Mann–Whitney U results support a dual strength model in the interpretation of this study: one of the key advantages of the direct technique is its fast implementation and aesthetics in the short term, while the semidirect technique excels in clinical endurance and quality retention over time. These statistical findings reinforce the clinical relevance of selecting restorative strategies based not only on immediate outcomes but also on the expected functional demands and longevity of the restoration.

## 4. Discussions

The comparison between the direct technique and the semidirect onlay technique for posterior composite restorations yielded important insights into their performance in terms of aesthetics, wear resistance, surface finish, marginal integrity, microleakage, and efficiency. The results showed that both techniques had unique strengths, but they also exhibited certain limitations based on the clinical situation and follow-up intervals. While the direct technique was initially superior in terms of aesthetic outcomes and patient satisfaction, the semidirect onlay technique demonstrated greater long-term durability and marginal integrity.

In the presented clinical case, for the direct technique, the application of a calcium hydroxide liner was not performed routinely but was based on case-specific clinical considerations. During cavity preparation, the patient experienced repeated episodes of pronounced hypersensitivity, which prompted a more conservative approach. Additionally, given the patient’s young age (18 years), the practitioner considered the likelihood of a relatively large pulp chamber based on the radiographic appearance. Although the remaining dentin thickness did not indicate an imminent pulp exposure, a protective liner was applied as a precautionary measure.

With respect to the use of caries detector dyes, it has to be emphasised that contemporary evidence increasingly supports selective caries excavation, while the routine use of caries detector solutions is controversial. In the present study, the caries detector was used only as an adjunctive visual aid in selected cases, particularly in deeper cavities, and not as a substitute for clinical judgement.

The aesthetic evaluation revealed that patients initially rated the direct technique higher due to the immediate visual replication of the natural occlusal anatomy. At T0, the aesthetic results were excellent, with minimal need for post-procedure adjustments. However, over time (T1–T3), slight discoloration and surface wear became more apparent in some cases, leading to a reduction in scores. Conversely, the semidirect onlay technique, while initially less aesthetic due to the added steps of polishing and adjustments, maintained a more stable appearance over time. The gradual wear observed in both techniques was a key factor influencing patient satisfaction, though it was more pronounced in restorations completed with the direct technique.

In terms of wear resistance, the semidirect onlay technique outperformed the direct technique. The greater wear resistance of semidirect onlays contributed to their longevity, making them more suitable for patients with higher occlusal loads. On the other hand, the direct technique, while faster and more efficient, exhibited more wear over time, especially in cases where the occlusal forces were higher.

In terms of efficiency, the direct technique is generally superior due to its simplicity and rapid execution. This method allows clinicians to replicate the occlusal anatomy quickly and with minimal need for post-procedure adjustments, significantly reducing chairside time. The ability to apply the composite directly in one visit, without the need for external fabrication, makes the direct technique ideal for cases where time is a critical factor. Conversely, while the semidirect onlay technique provides greater durability and precision, it is more time-consuming, requiring additional steps such as onlay fabrication outside the mouth and cementation, which extends the procedure time. As a result, for cases prioritising speed and efficiency, the direct technique offers a more streamlined and time-effective solution.

In terms of marginal integrity, the semidirect onlay technique tends to outperform the direct technique. This is primarily because the semidirect technique allows for better control over the adaptation of the composite material to the cavity margins. Since the onlay is fabricated outside the mouth, the material is cured under optimal conditions, reducing the risk of polymerization shrinkage that can compromise marginal integrity. Additionally, the ability to fine-tune the restoration outside the oral cavity ensures a more precise fit and tighter seal against the tooth structure, reducing the chances of microleakage. On the other hand, while the direct technique is faster and provides good initial results, it is more prone to slight marginal discrepancies due to the direct placement of the composite, especially in larger restorations where achieving perfect marginal adaptation can be challenging.

The direct technique is advantageous for its promptness, ease of application, and ability to replicate the natural occlusal anatomy with minimal adjustments, making it ideal for quick, aesthetic restorations [60]. However, it is more susceptible to wear and microleakage over time, especially if not executed precisely [15]. It is also less suitable for larger cavities where significant structural damage has occurred [61]. In contrast, the semidirect onlay technique offers better control, resulting in superior wear resistance and marginal integrity [62], but is more time-consuming due to the additional steps of external fabrication and cementation, potentially leading to greater patient discomfort.

For clinical cases where aesthetics are the primary concern, and the lesion is relatively small or moderate in size, the direct technique may be preferred [12]. Its ability to replicate the natural tooth anatomy quickly and with minimal post-procedure adjustments makes it ideal for patients [63] seeking fast, aesthetically pleasing results. This technique is particularly suitable for younger patients or those with intact occlusal surfaces, where the focus is on maintaining the natural contours and reducing chair time [12,42].

On the other hand, the semidirect onlay technique is more suitable for cases where durability is a priority, such as in patients with high occlusal forces [64] or larger cavities requiring a stronger restoration [55]. It is also preferred in situations where the tooth structure is more compromised [65], as the technique allows for better marginal adaptation and reduced risk of microleakage over time [66]. Additionally, for endodontically treated teeth or those with significant structural loss, the semidirect onlay provides better long-term stability [19].

One limitation of this study was the relatively short follow-up period of two years. While this timeframe provided valuable insights into the intermediate-term performance of the restorations, a longer follow-up period would be necessary to fully understand the long-term durability and success of both techniques. Additionally, the study excluded patients with bruxism or severe parafunctional habits, which could have provided a more comprehensive evaluation of wear resistance under extreme conditions. Furthermore, the study was conducted in a single dental clinic, which may limit the generalizability of the findings across different populations and settings.

Another limitation was the reliance on patient-reported outcomes for aesthetic satisfaction. While patient feedback is crucial, it is inherently subjective, and a more objective evaluation method, such as using advanced imaging techniques to measure colour stability and translucency, could enhance the reliability of the aesthetic assessments. Finally, while the study focused on two specific techniques, it did not account for variations in material selection or operator experience, which could influence the outcomes.

In a two-year randomised controlled trial, Elmoselhy et al. found that CAD/CAM-fabricated nano-hybrid composite onlays performed comparably to lithium disilicate onlays in restoring mutilated vital teeth, with both materials demonstrating acceptable clinical outcomes over the study period [67]. In contrast to the superior long-term performance observed in ceramic onlays over a five-year period, such as in the previous study and the one conducted by Yurdagüven et al. [68], the present two-year comparative analysis of composite restorations demonstrates that while the semidirect technique offers enhanced durability relative to the direct method, both composite approaches remain less predictable over extended periods, particularly in high-stress posterior applications.

When compared to the present study evaluating semidirect composite onlays, the findings from Gözetici-Çil et al. [69] further support the clinical viability of indirect composite restorations. Their three-year survival rate of 93.8% for partial indirect resin composite (PIRC) restorations, particularly those with partial cuspal coverage (i.e., onlays), aligns closely with the favourable functional outcomes observed in our semidirect group over a two-year period, specifically in terms of wear resistance, marginal integrity, and overall aesthetic retention. Both studies underscore the benefits of indirect placement and extraoral polymerization, which enhance marginal adaptation and reduce polymerization shrinkage.

Elhagrasy et al. [70] reported satisfactory marginal integrity and wear resistance for both conservative and conventional ceramic onlays after one year, whereas the present study extends these findings by demonstrating sustained performance of semidirect composite onlays over a two-year period.

In contrast to the in vitro findings of Ari et al. [71], which showed no statistically significant difference in microleakage between the stamp and conventional techniques, the present clinical study identified a clear long-term advantage in marginal integrity for semidirect composite onlays over direct restorations. While Ari et al. noted a higher percentage of severe leakage scores in the direct group (86.7%), our two-year clinical data revealed that semidirect onlays significantly outperformed the direct technique in marginal integrity at T3 (2.75 vs. 1.95), highlighting the importance of extraoral polymerization and precise marginal adaptation under clinical conditions.

An important factor contributing to the superior long-term performance of semidirect onlays is the adhesive cementation protocol [72]. Cementation was performed using resin-based cements such as Multilink Automix, RelyX Ultimate, or Nexus 3, which provide strong micromechanical and chemical bonding to enamel, dentin, and the indirect composite. Meticulous isolation and cavity cleaning were essential; therefore, a 2% chlorhexidine solution (Consepsis, Ultradent) was applied before and after 37% phosphoric-acid etching to reduce microbial load and enhance the durability of the adhesive layer [73]. Selective enamel etching was preferred because it increases adhesive strength and overall retention [74].

Resin cements also improve fracture resistance by minimising polymerization stresses [75]. Since the composite is fully polymerized extraorally, the restoration does not undergo shrinkage in situ, reducing the C-factor and stress on cavity walls [76]. The adhesive interface created by adhesive cementing systems such as Multilink Automix forms a monolithic tooth–restoration complex that distributes masticatory forces more evenly and decreases cusp deflection [72,75]. Studies by Pallesen & Van Dijken [77], Al-Haj Husain et al. [78], and Soares et al. [79] consistently show that adhesive resin cements significantly increase the fracture resistance of indirect composite onlays, often approaching values seen in intact teeth.

The resin cement layer also functions as a stress-absorbing buffer and ensures superior marginal sealing [53,77,78,79,80]. Adhesively cemented indirect composite restorations demonstrate lower microleakage and better long-term stability than those luted with non-adhesive materials [55,81]. Long-term data, such as the nine-year follow-up by Galiatsatos et al. [82], confirm high survival rates and durable performance of indirect composite onlays when bonded with resin cements.

Although the present study was not designed to establish rigid clinical indications or to stratify restorative performance according to cavity class, the inclusion of both Class I and Class II cavities reflects a pragmatic, real-world clinical approach consistent with daily restorative practice. Both direct composite restorations and semi-direct composite onlays are routinely indicated for posterior cavities of varying configurations, and the primary objective of this investigation was to compare the clinical performance of these techniques as applied in routine clinical decision-making rather than under highly standardised experimental conditions. Nevertheless, it must be acknowledged that differences in cavity geometry, remaining tooth structure, and load distribution may influence biomechanical behaviour and potentially affect clinical outcomes. Consequently, future prospective studies focusing on comparable cavity types—such as large Class II lesions restored using both direct and semi-direct approaches—may provide additional insight and allow for a more controlled assessment of technique-specific performance while minimising potential confounding factors.

In the present study, both direct composite restorations and semi-direct composite onlays were applied in Class I and Class II cavities, with the selection of technique based on cavity morphology, remaining tooth structure, and clinical indication rather than cavity class alone. Within the direct restoration group, the stamp technique was selectively employed when occlusal morphology could be predictably transferred, irrespective of whether the cavity was Class I or Class II. Detailed information regarding cavity size distribution, cavity type, and the type of restoration performed for each case is provided in a supplementary table (Supplementary Materials) accessible through the data repository link. The authors intentionally chose not to include this table in the main manuscript in order to avoid excessive length and to maintain clarity and focus on the predefined study objectives. The repository table contains additional descriptive parameters related to the treated cases, including cavity classification and restoration type, which were not considered primary variables in the study design but are made available to ensure transparency and allow interested readers to further explore the clinical dataset.

Based on the strengths of both techniques, a novel hybrid approach could combine the aesthetic and time efficiency of the direct technique with the durability and marginal integrity of the semidirect onlay. This hybrid technique would aim to optimise both functional and aesthetic outcomes by leveraging the benefits of both methods.

The first step in this hybrid approach would involve taking an impression of the intact occlusal anatomy, similar to the stamp technique. However, instead of directly applying the composite material, the impression would be used to create a customised onlay outside the mouth. The onlay would be fabricated with the same high-precision technique used in the semidirect onlay method, allowing for controlled curing and material handling. This step ensures that the restoration maintains optimal wear resistance and marginal integrity.

Once the onlay is prepared, it can be bonded to the tooth using the same adhesive techniques as the semidirect onlay method. The advantage here is that the occlusal anatomy is already replicated perfectly, reducing the need for further occlusal adjustments. This hybrid approach minimises the time spent in the chair while maximising the durability of the restoration.

The final step involves light curing and polishing the onlay, ensuring a smooth surface finish and excellent aesthetic integration with the adjacent teeth. By combining the efficiency and aesthetic benefits of the stamp technique with the long-term durability of the semidirect onlay, this hybrid technique could offer an optimal solution for a wide range of clinical situations. It would be especially useful in cases where patients desire both fast results and long-term success, without compromising on aesthetics or functionality.

The development of a hybrid technique could potentially address the limitations of both the direct and semidirect onlay techniques, providing a versatile and efficient approach to posterior composite restorations.

## 5. Conclusions

This clinical study provides a comprehensive comparative analysis of two restorative approaches for posterior composite restorations, the direct technique and the semidirect onlay technique, evaluated over a two-year period. The results demonstrate that while the direct technique offers significant advantages in terms of immediate aesthetic outcomes and procedural efficiency, its clinical performance tends to decline over time, particularly in aspects such as surface finish, wear resistance, and marginal integrity. In contrast, the semidirect onlay technique, though more time-intensive, consistently outperforms the direct method in long-term functional parameters, offering enhanced durability, superior marginal adaptation, and better resistance to occlusal wear.

Based on the findings, the direct technique is best suited for conservative restorations where the time is limited and immediate aesthetic integration are prioritised, especially in cases involving minimal structural loss. Meanwhile, the semidirect onlay technique proves more appropriate for extensive restorations or patients with high occlusal loads, where long-term clinical stability is essential.

Indirect composite onlays achieve better marginal integrity because polymerization occurs extraorally under controlled conditions, eliminating the shrinkage that compromises margins in direct techniques. The restoration is finished and adapted precisely before cementation, and the resin cement fills micro-defects, creating a continuous, sealed interface and increases the fracture resistance of indirect composite onlays. In contrast, direct techniques cure the composite intraorally, where polymerization shrinkage, cavity geometry, and potential contamination make it harder to achieve a perfectly sealed margin. This explains the consistently superior marginal closure of semidirect and indirect composite onlays compared to direct stamp restorations.

Given the complementary advantages of the two techniques, it is clear that there is a need for a novel hybrid restorative approach that merges the strengths of both methods to overcome their respective limitations. The proposed technique begins with the creation of a detailed occlusal imprint (stamp) of the patient’s intact tooth anatomy before cavity preparation. Instead of using this stamp intraorally, as in the conventional direct technique, the imprint serves as a negative template for fabricating a customised composite onlay extraorally. This allows for precise anatomical reproduction and improved control over polymerization, surface finishing, and marginal adaptation under ideal conditions outside the oral cavity.

## Figures and Tables

**Table 1:** Comparative analysis of semidirect (onlay) and direct restorative techniques across clinical parameters over time.

Criteria	Moment	Statistic	Semi-Direct Technique	Direct Technique
Aesthetics	T0	Mean	4.661691542288557	5.0
		Median	5.0	5.0
		Std.	0.6670645578788531	0.0
	T1	Mean	4.72636815920398	4.8979591836734695
		Median	5.0	5.0
		Std.	0.5382854668120761	0.303736860644865
	T2	Mean	4.582089552238806	4.625850340136054
		Median	5.0	5.0
		Std.	0.4944467736170386	0.4855569370242284
	T3	Mean	4.189054726368159	4.217687074829932
		Median	4.0	4.0
		Std.	0.39252974663081247	0.41408434722000503
Time and Efficiency	T0	Mean	2.4925373134328357	4.340136054421769
		Median	3.0	4.0
		Std.	0.735658908632762	0.6247914571471445
Surface Finish	T0	Mean	5.0	4.6938775510204085
		Median	5.0	5.0
		Std.	0.0	0.6580611298785054
Surface Finish	T1	Mean	4.9950248756218905	4.523809523809524
		Median	5.0	5.0
		Std.	0.0705345615858596	0.6551516956341014
	T2	Mean	4.9950248756218905	4.0476190476190474
		Median	5.0	4.0
		Std.	0.0705345615858596	0.6857985379856066
	T3	Mean	4.855721393034826	3.7755102040816326
		Median	5.0	4.0
		Std.	0.35224934633019434	0.4796676698770727
Wear Resistance	T1	Mean	4.751243781094527	4.238095238095238
		Median	5.0	5.0
		Std.	0.4667022019164169	1.1781883779265854
	T2	Mean	4.487562189054726	4.217687074829932
		Median	5.0	4.0
		Std.	0.5754081398131108	0.8400669151651454
	T3	Mean	3.8059701492537314	3.2993197278911564
		Median	4.0	3.0
		Std.	1.047456051156552	1.1251481849826666
Marginal Integrity and Microleakage	T1	Mean	4.641791044776119	4.2517006802721085
		Median	5.0	4.0
		Std.	0.5839903904341264	0.912947487686727
	T2	Mean	3.890547263681592	2.9115646258503403
		Median	4.0	3.0
		Std.	1.134883341584048	1.2976542063616345
	T3	Mean	2.746268656716418	1.945578231292517
		Median	3.0	2.0
		Std.	1.3001148054932257	1.115165157159268

**Table 2:** Repeated-Measures ANOVA results showing changes across the measured time points.

Criterion	F-Value	Numerator DF	Denominator DF	*p*-Value
Aesthetic	180.3	3	1041	<0.0001
Surface Finish	163.66	3	1041	<0.0001
Wear Resistance	137.93	2	694	<0.0001
Marginal Integrity	642.79	2	694	<0.0001

**Table 3:** Wilcoxon Signed-Rank Test Results showing statistical comparisons between adjacent timepoints for each criterion.

Criterion	Comparison	W-Statistic	*p*-Value
Aesthetic	T0 vs. T1	490.5	0.955005
	T1 vs. T2	867	1.65 × 10^−11^
	T2 vs. T3	0	4.4 × 10^−32^
Surface Finish	T0 vs. T1	143.5	3.94 × 10^−5^
	T1 vs. T2	36.5	1.59 × 10^−16^
	T2 vs. T3	73	1.11 × 10^−15^
Wear Resistance	T1 vs. T2	7625	0.000765
	T2 vs. T3	0	4.22 × 10^−32^
Marginal Integrity	T1 vs. T2	0	4.65 × 10^−38^
	T2 vs. T3	0	9.33 × 10^−40^

**Table 4:** Mann–Whitney U test results, showing statistical comparisons between the Semidirect and Direct techniques for each timepoint and criterion.

Criterion	Timepoint	U-Statistic	*p*-Value
Aesthetic	T0	11,392.5	5.62 × 10^−10^
	T1	12,832.5	0.001518
	T2	14,127	0.41138
	T3	14,350.5	0.511543
Surface Finish	T0	17,688	5.37 × 10^−11^
	T1	20,534.5	1.48 × 10^−21^
	T2	26,167.5	3.1 × 10^−50^
	T3	27,836	1.12 × 10^−55^
Wear Resistance	T1	17,639	0.000117
	T2	17,066.5	0.00591
	T3	18,656.5	1.28 × 10^−5^
Marginal Integrity	T1	18,261	1.42 × 10^−5^
	T2	21,040	3.55 × 10^−12^
	T3	20,032	4.74 × 10^−9^

## Data Availability

The data supporting the findings of this study are available from the corresponding author upon reasonable request. However, access to the full dataset is restricted due to privacy and confidentiality policies mandated by the Romanian Ministry of Defense, given the involvement of military personnel. The research was registered at Open Science Framework (OSF) and can be found under registration DOI doi.org/10.17605/OSF.IO/ZVTXU. All the metadata was uploaded and is available under the registration code osf.io/tvn8r. This repository includes the patient consent form, the clinical protocol provided to practitioners to ensure standardised application of each technique, and the evaluation questionnaire with detailed scoring criteria and guidance for its administration. This study does not constitute a clinical trial, as it involved no testing of new materials or techniques. All procedures analysed are well-established and routinely used in clinical practice. As an observational study with no experimental intervention, registration in a public clinical trial registry was not applicable, in accordance with international guidelines.

## Supplementary Materials

The following supporting information can be downloaded at: https://archive.org/details/osf-registrations-zvtxu-v1 (accessed on 12 January 2026). Patient consent form, clinical protocol and the Evaluation Questionnaire.
